# Duration of post–COVID-19 symptoms is associated with sustained SARS-CoV-2–specific immune responses

**DOI:** 10.1172/jci.insight.151544

**Published:** 2021-08-09

**Authors:** Jacob K. Files, Sanghita Sarkar, Tim R. Fram, Sushma Boppana, Sarah Sterrett, Kai Qin, Anju Bansal, Dustin M. Long, Steffanie Sabbaj, James J. Kobie, Paul A. Goepfert, Nathan Erdmann

**Affiliations:** 1 Division of Infectious Diseases, Department of Medicine, School of Medicine,; 2 Department of Biostatistics, School of Public Health, and; 3 Department of Microbiology, School of Medicine, University of Alabama at Birmingham, Birmingham, Alabama, USA.

**Keywords:** COVID-19, Immunology, Adaptive immunity

## Abstract

A subset of COVID-19 patients exhibit post-acute sequelae of COVID-19 (PASC), but little is known about the immune signatures associated with these syndromes. We investigated longitudinal peripheral blood samples in 50 individuals with previously confirmed SARS-CoV-2 infection, including 20 who experienced prolonged duration of COVID-19 symptoms (lasting more than 30 days; median = 74 days) compared with 30 who had symptom resolution within 20 days. Individuals with prolonged symptom duration maintained antigen-specific T cell response magnitudes to SARS-CoV-2 spike protein in CD4^+^ and circulating T follicular helper cell populations during late convalescence, while those without persistent symptoms demonstrated an expected decline. The prolonged group also displayed increased IgG avidity to SARS-CoV-2 spike protein. Significant correlations between symptom duration and both SARS-CoV-2–specific T cells and antibodies were observed. Activation and exhaustion markers were evaluated in multiple immune cell types, revealing few phenotypic differences between prolonged and recovered groups, suggesting that prolonged symptom duration is not due to persistent systemic inflammation. These findings demonstrate that SARS-CoV-2–specific immune responses are maintained in patients suffering from prolonged post–COVID-19 symptom duration in contrast to those with resolved symptoms and may suggest the persistence of viral antigens as an underlying etiology.

## Graphical abstract

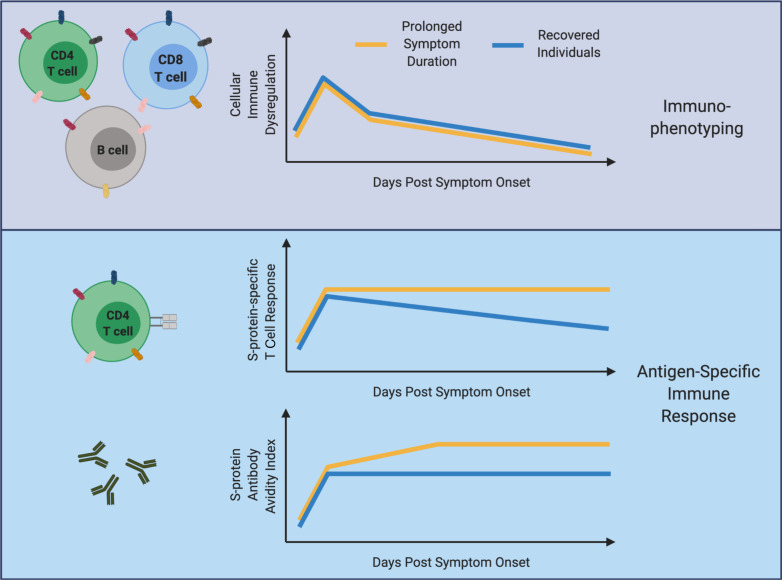

## Introduction

COVID-19, caused by the SARS-CoV-2 virus, has infected millions of individuals and caused profound morbidity and mortality worldwide. Our group and others have characterized the acute immune response to COVID-19, finding dramatic immune dysregulation in peripheral blood samples from infected individuals (1–7), especially in those with severe infection. Evidence suggests some of these immune perturbations persist into the convalescent phase of infection (1, 8, 9). Antigen-specific immune responses during the acute and early convalescent stages of infection have been found to play an important role in overall patient outcomes (10–15). Reports have shown that SARS-CoV-2–specific T cell memory is maintained for months after initial symptom onset in convalescent PBMC samples, but the magnitude of observed T cell responses decreases over time (14, 16–21). In contrast, numerous studies have detected increased magnitudes of IgG^+^ SARS-CoV-2–specific memory B cells in the blood of convalescent patients during late convalescence, suggesting that the memory B cell population is sustained in the months following acute infection (16, 18, 20, 22). Despite this, many groups have found that SARS-CoV-2–specific antibodies, and neutralizing antibodies in particular, decrease within the first few months following initial symptom onset in many individuals (16, 18–20, 22–24).

An emerging complication of COVID-19 infection is a prolonged period of symptoms involving multiple organ systems for months after the initial onset of symptoms in a subset of individuals (25–30). Similar long-term sequelae have been described for other viral illnesses, including Chikungunya and Ebola, as well as the coronaviruses SARS and Middle East respiratory syndrome (31–35). While the prevalence of symptoms following COVID-19 infection is not well-defined, numerous reports describe what is now being called post-acute sequelae of COVID-19 (PASC). The underlying immune mechanisms and pathophysiology of these syndromes remain unclear. A recent study detected SARS-CoV-2 RNA from intestinal biopsies taken from patients during the convalescent phase of infection 4 months after initial symptom onset, showing that SARS-CoV-2 viral antigen can persist in convalesced patients (22). Overall much remains unknown in regard to patients with PASC, especially in terms of immune dysregulation and overall immune memory formation.

In the current study, we comprehensively profiled longitudinal samples from convalescent patients to assess potential immune differences between individuals experiencing prolonged symptom duration and those with complete recovery. Our results show few differences in systemic phenotyping of various immune subsets between prolonged and recovered individuals. However, patients with prolonged symptom duration exhibited increased SARS-CoV-2 S-protein–specific antibody avidity and T cell responses that did not decline during the intermediate and late convalescent phases, respectively.

## Results

### Overview of patient cohort.

**Figure 1:**
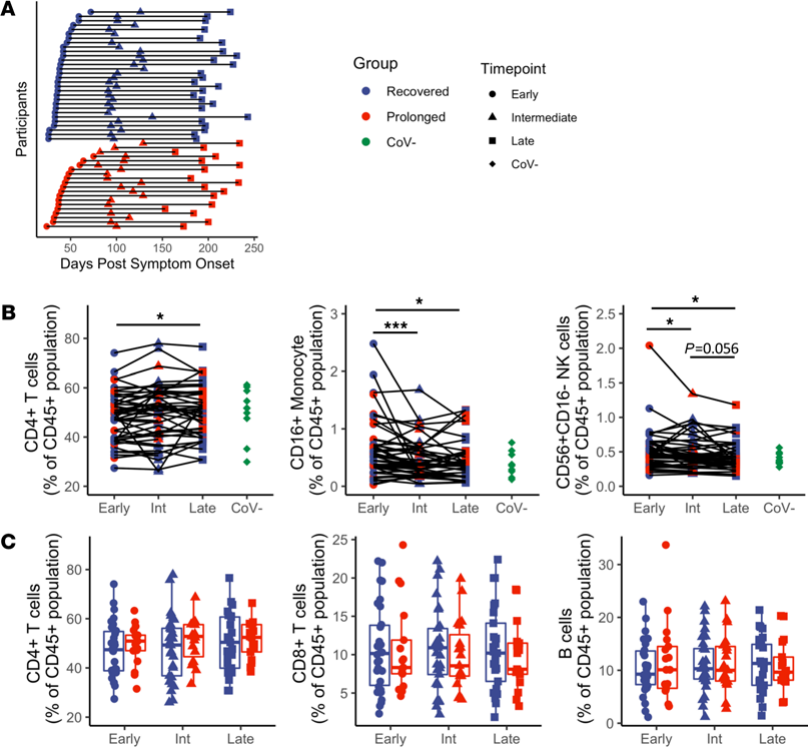
Immune cell subset frequencies show no difference between prolonged and recovered groups. (**A**) Cohort sampling overview of all participants (*n* = 50) by days after symptom onset. Immune cell subset phenotyping was performed on PBMC samples for all individuals. (**B**) Longitudinal analysis (*n* = 50) reveals differences in frequencies of CD4^+^ T cells, CD16^+^ monocytes, and CD56^+^CD16^–^ NK cells. (**C**) No differences were observed in frequencies of CD4^+^ T cells, CD8^+^ T cells, or B cells when comparing recovered (*n* = 30) and prolonged (*n* = 20) groups. Navy = recovered group, red = prolonged group, green = CoV^–^; circles = early, triangles = intermediate, squares = late, diamonds = CoV^–^. Box plots indicate median, IQR, and 95% confidence interval; significance determined by the paired Wilcoxon’s signed-rank (**B**) or the unpaired Wilcoxon’s rank sum tests (**C**) and indicated as follows: *P ≤ 0.05, **P ≤ 0.01, ***P ≤ 0.001.

Prior studies have provided an overview of the phenotypic changes that occur following SARS-CoV-2 infection (1, 17), but whether differences in symptom duration are associated with immunologic alterations has yet to be defined. Here, we analyzed peripheral blood samples at longitudinal time points from a total of 50 individuals with confirmed COVID-19 infection by either PCR or antibody testing. An overview of this cohort is shown in Table 1, with additional clinical information provided in Supplemental Table 1; supplemental material available online with this article; https://doi.org/10.1172/jci.insight.151544DS1 In this cohort, 30 patients recovered from their initial COVID-19 symptoms with no residual complaints (median symptom duration = 10 days; range 1–20). The remaining 20 patients had documented symptoms for at least 30 days (median symptom duration = 73.5 days; range: 30–208). Although PASC does not yet have a strict definition, the criteria applied here were consistent with other characterizations (25, 29, 30). The 20 patients experiencing symptoms for more than 30 days are referred to as the “prolonged” group, while the remaining 30 are the “recovered” group. Symptoms reported beyond 30 days in the prolonged group included dyspnea, fatigue, psychataxia, and/or cough. (Note: isolated anosmia/ageusia for more than 30 days did not meet our criteria for classification into the prolonged group.) As expected, the prolonged group had higher frequencies of hospitalization and severe infection (based on peak ordinal score), consistent with previous studies that have shown up to 76% of hospitalized patients reported at least 1 residual symptom at 6 months after initial symptom onset (27). Otherwise, the groups had minimal differences in age, race, and sex. Samples collected during the first 75 days following initial symptom onset were classified as “early” convalescence period, between 76 and 150 days following symptom onset as “intermediate” convalescence period, and after 151 days after symptom onset as “late” convalescence period. An overview of participants and their respective sample collection dates in relation to days after symptom onset is shown in Figure 1A. In addition to these 50 individuals, specimens from 10 healthy controls collected prior to the COVID-19 pandemic between 2014 and 2018 (CoV^–^ group) were included in the analyses.

Each participant had a confirmed positive test for SARS-CoV-2 during acute infection (excluding CoV^–^) by PCR or nucleocapsid antibody. Nasopharyngeal swabs were collected and frozen for 49 of 50 individuals at an additional time point during early or intermediate convalescence (median collection time point in days after symptom onset = 94 days; range: 34–153 days). All follow-up nasal swabs were negative for SARS-CoV-2 RNA, suggesting that differences in symptom duration were not due to persistence of SARS-CoV-2 RNA in the upper respiratory tract.

### Immune cell subset frequencies show no difference between prolonged and recovered groups.

Using flow cytometric analysis, our group first determined the relative frequencies of different immune cell subsets in the peripheral blood. We and others have previously shown cell subset frequencies remain altered into the convalescent phase after SARS-CoV-2 infection (1, 8), but few reports comprehensively address whether this persists into late convalescence or is associated with PASC. Here, cell frequencies were reported as a frequency of the overall CD45^+^ population per each individual as done previously (1). Subsample automated uniform manifold approximation and projection (UMAP) clustering of all CD45^+^ cells in the cohort demonstrated a total of 18 populations and is presented in Supplemental Figure 1, A and B.

Investigating longitudinal changes across the entire cohort (*n* = 50), we observed that CD4^+^ T cell frequencies increased between early and late convalescence (Figure 1B; *P* = 0.042). This may reflect a recovery from the acute lymphopenia associated with COVID-19 infection that our group and others have previously described (36–39). We also observed a decrease in the nonclassical CD14^–^CD16^+^ monocyte population over time (Figure 1B; *P* < 0.001 between early and intermediate time points; *P* = 0.026 between early and late time points). Similarly, there was a significant decrease in the CD56^+^CD16^–^ NK cell frequency between both early and intermediate time points (Figure 1B; *P* = 0.020) and between the early and late time points (Figure 1B; *P* = 0.016), as well as a trend toward decrease between the intermediate and late time points (Figure 1B; *P* = 0.056). We did not observe differences between prolonged and recovered groups in terms of CD4^+^ T cells, CD8^+^ T cells, or B cells (Figure 1C) and observed minimal differences between monocytes, NKT cells, and NK cells (Supplemental Figure 1, C and D). Overall, these immune cell populations returned to baseline following COVID-19 infection, suggesting that prolonged symptom duration is not associated with substantial systemic changes in immune cell subset frequencies.

### Prolonged and recovered groups have similar activation marker frequencies.

We next investigated the expression of various activation and exhaustion markers. Prior reports found decreased expression of HLA-DR on monocyte subsets in the setting of SARS-CoV-2, predominantly in individuals with severe COVID-19 infection (40, 41). Here, we observed no differences in HLA-DR expression between prolonged and recovered groups in monocyte subsets (classical CD14^+^CD16^–^ monocytes, nonclassical CD14^–^CD16^+^ monocytes, and intermediate CD14^+^CD16^+^ monocytes; Supplemental Figure 2). We also found no significant changes over time when investigating HLA-DR expression at different phases of convalescence in all 3 monocyte subsets.

**Figure 2:**
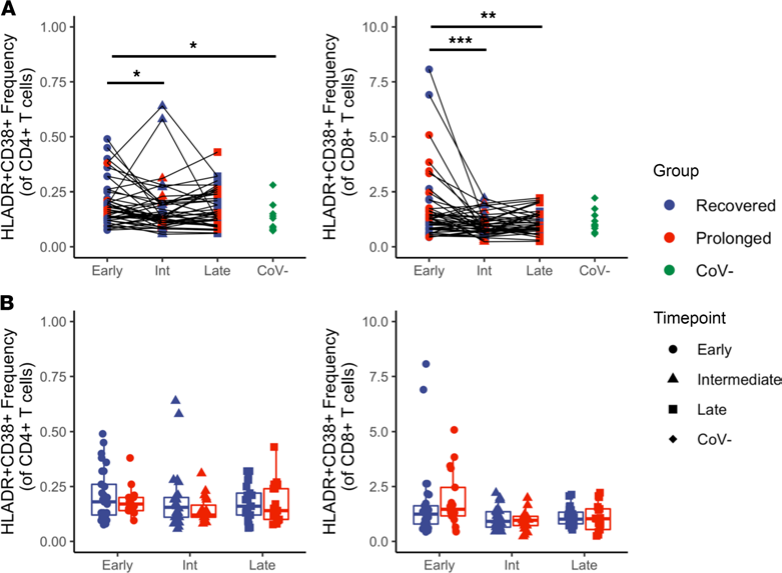
T cell activation during early convalescence recovers over time and shows no differences based on duration of symptoms. Investigation into the expression of HLA-DR and CD38 within CD4^+^ and CD8^+^ T cell subsets. (**A**) Comparison made longitudinally over time (*n* = 49). (**B**) Comparison between recovered (*n* = 29) and prolonged (*n* = 20) groups. Navy = recovered group, red = prolonged group, gree*n* = CoV^–^; circles = early, triangles = intermediate, squares = late, diamonds = CoV^–^. Box plots indicate median, IQR, and 95% confidence interval; significance determined by the paired Wilcoxon’s signed-rank (**A**) or the unpaired Wilcoxon’s rank sum tests (**B**) and indicated as follows: *P ≤ 0.05, **P ≤ 0.01, ***P ≤ 0.001.

We next investigated longitudinal phenotypic differences in T cells between those experiencing persistent symptoms and those with resolved disease. Our group and others have observed T cell activation that persists into the early convalescent phase following SARS-CoV-2 infection (1, 8), but few studies have investigated phenotypic differences into the intermediate and late phases of convalescence, particularly in individuals with prolonged symptom syndromes. One recent study observed longitudinal changes in T cell activation independent of ongoing symptoms (9). To investigate T cell changes in our cohort, we first characterized T cell activation as determined by dual expression of HLA-DR and CD38, previously shown to be upregulated in severely infected COVID-19 patients (2), and found that the CD4^+^ T cell population had increased HLA-DR^+^CD38^+^ expression when comparing early and intermediate time points, as well as when comparing the early time point with CoV^–^ controls (Figure 2A; *P* = 0.024 and *P* = 0.021, respectively). Similarly, we observed increased HLA-DR and CD38 dual expression in the CD8^+^ T cell subset during the early time point when compared with both the intermediate and late time points (Figure 2A; *P* < 0.001 and *P* = 0.007, respectively). Importantly, there were no significant differences in T cell expression of HLA-DR and CD38 between prolonged and recovered groups in either CD4^+^ or CD8^+^ T cell populations (Figure 2B). We found no differences in memory (CD28^+^CD57^-^) or senescent (CD28^–^CD57^+^) T cell populations for either CD4^+^ T cells (Supplemental Figure 3A) or CD8^+^ T cells (Supplemental Figure 4A). Overall, these data support our previous findings that immune activation persists into early convalescence, but this systemic dysregulation appears to resolve by the intermediate and late convalescent phase in both groups.

We evaluated the expression of additional T cell surface activation markers, including OX40, CD69, CD154, and CD137. The markers programmed cell death 1 (PD-1), TIGIT, TIM3, and programmed cell death ligand 1 (PD-L1) are traditionally thought to be exhaustion markers upregulated during chronic antigen exposure but have also been shown to have increased expression in several acute infections, including COVID-19 (6, 42, 43). Finally, we looked into the presence of effector and memory T cell populations by the expression of CD45RA and CCR7. A summary of our clusters following UMAP analysis is shown in Supplemental Figure 3B and Supplemental Figure 4B, where we identified 10 different populations in both CD4^+^ and CD8^+^ T cell subsets. Overall, we found no evidence to suggest differences in activation or exhaustion in either CD4^+^ T cells (Supplemental Figure 3, C and D) or CD8^+^ T cells (Supplemental Figure 4, C and D). We did observe minor differences within the central memory (CD45RA^–^CCR7^+^) T cell population with increased frequencies in individuals with prolonged symptom duration at all time points (Supplemental Figure 3E; early: *P* = 0.027; intermediate: *P* = 0.048; late: *P* = 0.029). We did not observe any significant differences in effector or memory CD8^+^ T cell populations (Supplemental Figure 4E).

### Convalescent COVID-19 patients with prolonged symptom duration exhibit maintained SARS-CoV-2–specific T cell response magnitudes.

**Figure 3:**
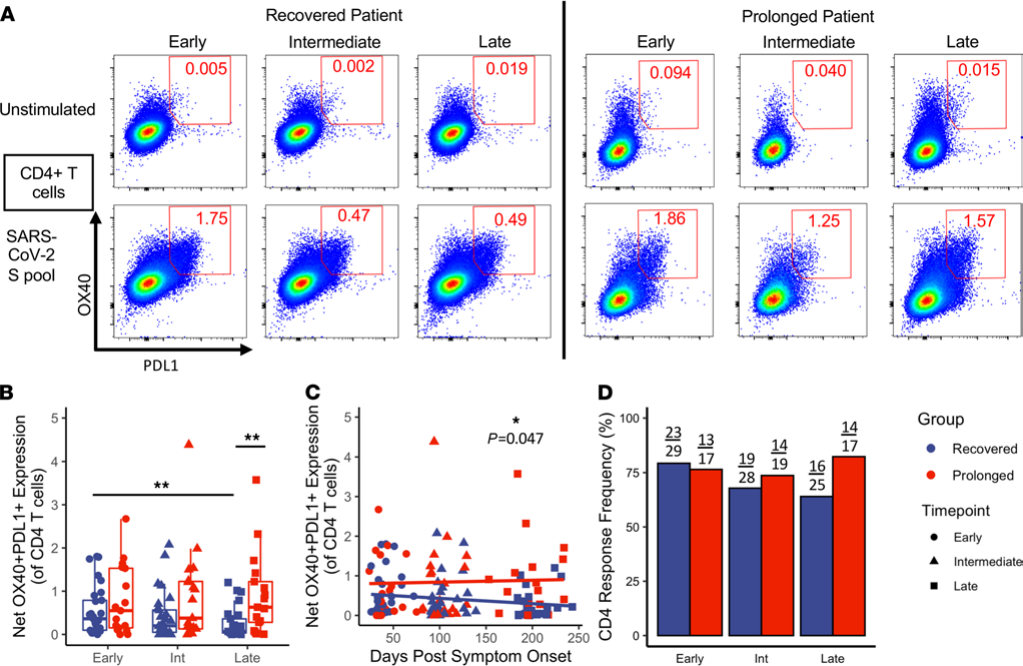
Maintenance of S-protein–specific CD4^+^ T cell response magnitude in individuals with prolonged symptom duration. Investigation into the upregulation of OX40 and PD-L1 in CD4^+^ T cells after stimulation with SARS-CoV-2 S pool. (**A**) Representative flow cytometry plots of a recovered and a prolonged individual at all 3 time points. (**B**) Comparisons of the net CD4^+^ T cell response magnitude between recovered (*n* = 29) and prolonged (*n* = 20) groups; significance between time points determined by paired Wilcoxon’s signed-rank test and significance between groups determined by unpaired Wilcoxon’s rank sum. (**C**) Comparisons of the net CD4^+^ T cell response magnitude made longitudinally by DPSO (*n* = 135 across all 3 time points); significance determined by linear mixed effects modeling. (**D**) Frequencies of CD4^+^ T cell responses meeting positivity criteria; no significance determined by Fisher’s exact. Navy = recovered group, red = prolonged group; circles = early, triangles = intermediate, squares = late. Box plots indicate median, IQR, and 95% confidence interval; significance is indicated as follows: *P ≤ 0.05, **P ≤ 0.01, ***P ≤ 0.001.

After observing minimal phenotypic differences between prolonged and recovered groups, we assessed antigen-specific T cell memory responses. Several groups have described the longitudinal nature of T cell memory in patients recovering from SARS-CoV-2 infection (16–19). These reports demonstrate that T cell responses persist 6 months out from initial symptom onset, but response magnitude wanes over time. Here, we utilized an activation-induced marker (AIM) assay previously described to detect antigen-specific responses in multiple T cell subsets with high sensitivity (16, 18, 44, 45). In brief, after stimulation with pooled sequence-matched SARS-CoV-2 spike protein peptides (SARS-CoV-2 S pool), we investigated antigen-specific upregulation of surface activation markers. To assess antigen-specific CD4^+^ T cell responses, we measured the upregulation of OX40 and PD-L1; representative longitudinal examples are shown in a recovered and a prolonged patient in Figure 3A.

In the recovered group, we observed a decrease in CD4^+^ T cell response magnitude to SARS-CoV-2 S pool between the early and late time points (Figure 3B; *P* = 0.006; analysis shown in a paired manner in Supplemental Figure 5A). However, a similar decrease in response magnitude was not observed in the prolonged symptom group. Individuals in the prolonged group had a higher CD4^+^ T cell response magnitude at the late convalescent time point compared with the recovered group (Figure 3B; *P* = 0.007). When looking at how CD4^+^ T cell responses change based on DPSO, similar relationships were observed (Figure 3C). By using a mixed effects linear model that considers multiple time points for each individual patient, the model for the recovered group had a slope of –0.00147, indicating a decay in net CD4 response magnitude by 0.00147 every day; over the course of the 243-day study, this would correspond to a net decrease in 0.357. In contrast, the prolonged group had a slope of 0.00038. The mixed effects linear model found that these 2 slopes were significantly different (Figure 3C; *P* = 0.047). A majority of the individuals showed positive CD4 responses at all time points based on our positivity criteria (Figure 3D). Thus, despite the decrease in response magnitude observed in the recovered group, SARS-CoV-2–specific CD4^+^ T cells were still detectable during late convalescence. These data are consistent with prior reports that observed a decreased CD4^+^ T cell response magnitude over time following quick resolution of COVID-19 symptoms (16, 18). However, to our knowledge, this is the first report that patients with prolonged symptom duration maintain CD4^+^ T cell response magnitudes up to 6 months following initial symptom onset.

**Figure 4:**
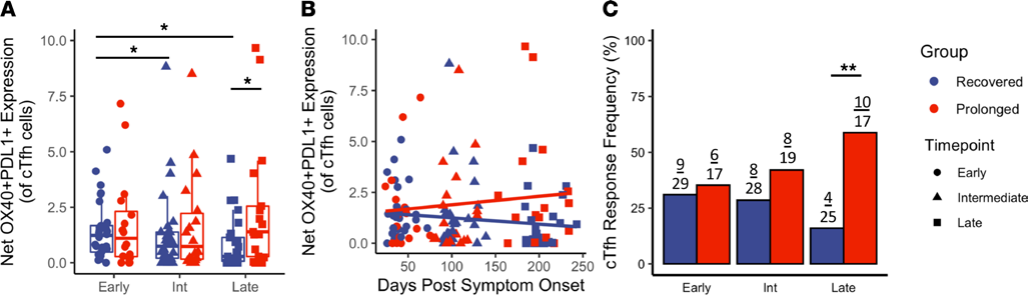
Persistent S-protein–specific cTfh T cell responses in individuals with prolonged symptom duration. Investigation into the upregulation of activation markers in the cTfh (CD4^+^CXCR5^+^PD-1^+^) subset. (**A**) Comparisons of the net cTfh OX40^+^PD-L1^+^ response magnitude between recovered (*n* = 29) and prolonged (*n* = 20) groups; significance between time points determined by paired Wilcoxon’s signed-rank test and significance between groups determined by unpaired Wilcoxon’s rank sum. (**B**) Comparisons of the net cTfh T cell response magnitude longitudinally by DPSO (*n* = 135 across all 3 time points); no significance determined by linear mixed effects modeling. (**C**) Frequencies of cTfh T cell responses meeting positivity criteria; significance determined by Fisher’s exact. Navy = recovered group, red = prolonged group; circles = early, triangles = intermediate, squares = late. Box plots indicate median, IQR, and 95% confidence interval; significance is indicated as follows: *P ≤ 0.05, **P ≤ 0.01, ***P ≤ 0.001.

We next investigated the presence of circulating T follicular helper cells (cTfhs). Human cTfhs have been observed in response to various infections and vaccinations. In the setting of HIV-vaccination, cTfhs have been shown to be clonal derivatives of T follicular helper cells found in lymphoid tissue, and increased presence of cTfhs correlates with HIV-specific antibodies (46). Our group has also described that an improved cTfh response leads to enhanced opsonophagocytic antibody responses in younger individuals given a conjugated pneumococcal vaccine (47). In this study, we define cTfh cells as those CD4^+^ T cells expressing CXCR5 and PD-1. When looking at the overall frequencies of cTfhs, we observed no differences between groups or between time points (Supplemental Figure 5B). We next quantified the SARS-CoV-2–specific cTfh populations, by identifying the antigen-specific upregulation of OX40 and PD-L1; a representative example is shown in Supplemental Figure 5C. In the recovered group, a decreased response magnitude was observed when comparing the early time point with the intermediate and late time points (Figure 4A; *P* = 0.047 and *P* = 0.011, respectively; analysis shown in a paired manner in Supplemental Figure 5D). However, similar to the CD4^+^ population, a sustained cTfh antigen-specific response magnitude was observed in prolonged individuals, with a significant difference in late convalescence when comparing cTfh response magnitude between prolonged and recovered groups (Figure 4A; *P* = 0.045). Comparing the cTfh response magnitude by DPSO yielded similar slopes to the antigen-specific T cell population at large, with the recovered individuals decreasing at 0.0034 per day. However, we observed a slight increase in cTfh response magnitude in the prolonged group at later time points (Figure 4B; no significant difference between slopes). Interestingly, we observed a decrease in the overall number of cTfh responses that met our positive criteria in the recovered group (9 of 29 decreasing to 4 of 25) but found an increase in the overall number of cTfh responses in individuals with prolonged symptom duration (6 of 17 increasing to 10 of 17); this resulted in a significant difference in cTfh responder frequency between groups at the late time point (Figure 4C; *P* = 0.007). These data further support the observed increase in antigen-specific activation of the T cell populations during late convalescence in individuals with prolonged symptom duration following COVID-19 infection.

We also investigated antigen-specific CD8^+^ T cell responses as previously demonstrated by assessing the upregulation of CD69 and CD137 (16, 18). Overall, we observed a lower magnitude and frequency of CD8^+^ T cell responses than previous reports investigating convalescent COVID-19 individuals, which is likely attributable to the use of 17-mer peptides for the S-protein only. Despite this, we observed similar trends to CD4^+^ T cell and cTfh responses at the late convalescent time point with a trend toward increased CD8^+^ T cell response magnitude in individuals with prolonged symptom duration (Supplemental Figure 6A; *P* = 0.095). In the recovered group, there appeared to be a decrease in the CD8^+^ T cell response magnitude over time, while the response magnitude was maintained longitudinally in the prolonged group (Supplemental Figure 6B; no significant difference between slopes). Finally, there was a trend toward increased CD8^+^ T cell response frequency in the prolonged group at the late time point, with 3 of 17 individuals showing a detectable CD8^+^ T cell response, while there were 0 of 25 individuals with a CD8^+^ T cell response in the recovered group (Supplemental Figure 6C; *P* = 0.059). Collectively, these data show that individuals with a prolonged symptom duration appear to have a relative increase in T cell response magnitude at the late convalescent time point.

### Increased SARS-CoV-2 S-protein–specific IgG avidity in patients with prolonged symptom duration.

**Figure 5:**
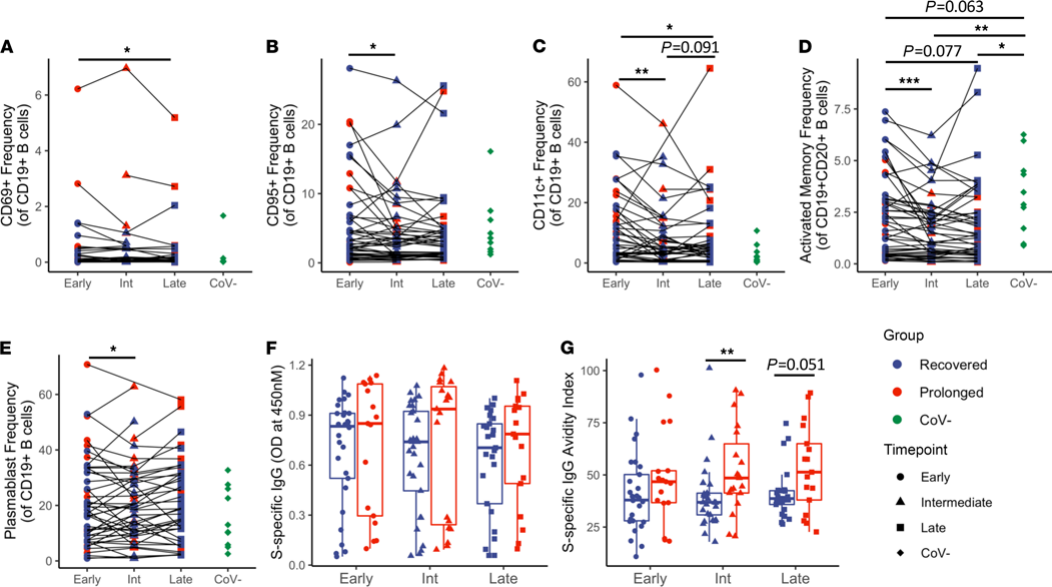
B cell phenotyping and IgG antibody data show increased S-protein specific IgG avidity in prolonged symptom group. (**A**–**C**) Longitudinal analysis (*n* = 50) of B cell phenotype by activation markers CD69, CD95, and CD11c, respectively. (**D** and **E**) Longitudinal analysis (*n* = 50) of activated memory and plasmablast B cell subsets. (**F**) Longitudinal analysis of S-specific sera IgG from recovered (*n* = 30) and prolonged (*n* = 20) groups as determined by ELISA. (**G**) Avidity index of S-protein–specific IgG determined by ELISA with or without 8 M urea treatment. Navy = recovered group, red = prolonged group, green = CoV^–^; circles = early, triangles = intermediate, squares = late, diamonds = CoV^–^. Box plots indicate median, IQR, and 95% confidence interval; significance determined by the paired Wilcoxon’s signed-rank (**A**–**E**) or the unpaired Wilcoxon’s rank sum tests (**F**–**G**) and are indicated as follows: *P ≤ 0.05, **P ≤ 0.01, ***P ≤ 0.001.

Our group and others have reported perturbations in peripheral blood B cell subsets during acute COVID-19 infection, with some persisting into early convalescence (1, 2, 7, 15). Examination of B cells for expression of activation- and exhaustion-related markers and major B cell subsets did not reveal substantial differences between prolonged and recovered groups (Figure 5 and Supplemental Figure 7). For the entire cohort, a general longitudinal decrease in activated B cell populations (CD69^+^, CD95^+^, CD11c^+^, and CD21^–^CD27^+^ activated memory) was evident (Figure 5, A–D). A longitudinal decrease in plasmablasts was also observed (Figure 5E), consistent with resolution of the expansion of plasmablasts that has been previously reported during acute COVID-19 infection (2, 7, 15). In both prolonged and recovered groups, there is a longitudinal trend toward return to B cell homeostasis following COVID-19 infection.

We next sought to determine if the features of the plasma binding antibody response to SARS-CoV-2 were altered in the prolonged group. A trend toward increased S-specific IgG in the prolonged group was evident, which was more pronounced in the intermediate and late time points (Figure 5F). Using 8 M urea, a chaotropic agent to reduce low-avidity IgG binding and a surrogate for measuring affinity maturation, the prolonged group developed higher avidity S-specific IgG at each time point and reached statistical significance compared with the recovered group at the intermediate time point (Figure 5G; *P* = 0.009). No significant differences in the SARS-CoV-2 nucleocapsid-specific (N-specific) plasma IgG response were apparent between the groups (data not shown). These results suggest that increased affinity maturation of the SARS-CoV-2 antibody response occurs in patients with prolonged symptom duration.

### Magnitude of SARS-CoV-2–specific immune response correlates to symptom duration.

**Figure 6:**
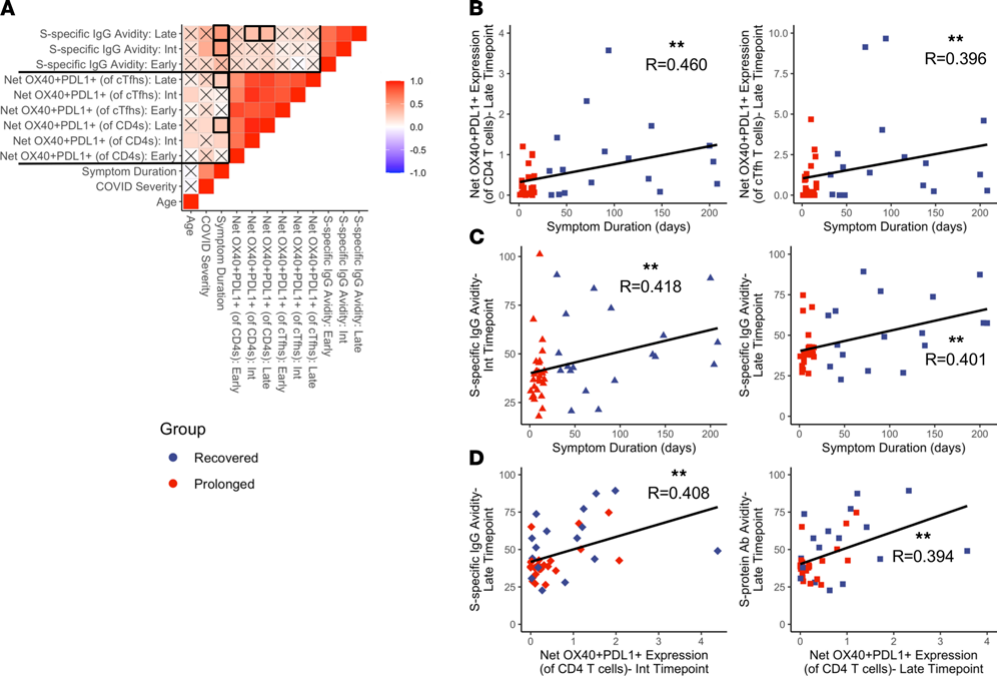
Symptom duration correlates with late antigen-specific T cell responses and S-specific antibody avidity. Investigation into correlations between symptom duration, antigen-specific T cell responses, and S-protein–specific antibody avidity. (**A**) Correlogram showing all correlations between chosen variables. Statistical analyses were performed using Spearman’s correlation test; *X*s denote correlations with nonsignificant *P* values, and color denotes the correlation coefficient. Bolded outlines indicate relevant correlations, which are emphasized in panels **B**–**D**. (**B**) Correlation graphs between symptom duration and S-protein–specific CD4^+^/cTfh subsets at the late time point. (**C**) Correlation graphs between symptom duration and S-protein–specific antibody avidity index at the intermediate and late time points. (**D**) Correlation graphs between S-protein–specific antibody avidity index at the late time point and S-protein–specific CD4^+^ T cell responses at the intermediate and late time points. Navy = recovered group, red = prolonged group; triangles = intermediate time point, squares = late time point, diamonds = mismatched time points. Significance determined by Spearman’s rank-order correlation and denoted as follows: *P ≤ 0.05, **P ≤ 0.01, ***P ≤ 0.001.

Finally, we assessed correlative relationships between our different demographic and immune variables. A correlogram showing various demographic and antigen-specific variables is presented in Figure 6A. The correlogram shows the most significant and strongest correlations between antigen-specific CD4^+^ T cell and cTfh responses to the S-protein across all time points, indicating that the magnitude of CD4^+^ T cell responses correlates to the magnitude of cTfh responses. Similarly, there were significant correlations between S-protein antibody avidity index across different time points. Notably, we found significant correlations at the late time point between symptom duration and net CD4^+^ T cell response magnitude as well as between symptom duration and net cTfh T cell response magnitude (Figure 6B; *P* = 0.002 and *P* = 0.009, respectively). Symptom duration also correlated with the S-protein antibody avidity, both at the intermediate and late time points (Figure 6C; *P* =0.003 and *P* = 0.008). These data further support the observation that individuals with prolonged symptom duration exhibit increased maintenance of SARS-CoV-2–specific immunity in late convalescence.

We also investigated whether CD4^+^ T cell responses may have affected the generation of S-specific antibodies. Individuals with increased net OX40^+^PD-L1^+^CD4^+^ T cell responses at the intermediate and late time points showed higher avidity S-specific IgG at the late time point (Figure 6D; *P* = 0.009 and *P* = 0.010, respectively). There was also a trend between S-specific IgG avidity index and net cTfh response magnitude (data not shown; *P* = 0.051). Interestingly, we also observed strong correlations between antigen-specific CD4 responses at all time points and overall S-specific IgG binding at all time points. Correlation plots showing the S-specific IgG and representative CD4 and cTfh responses from the early time point are shown in Supplemental Figure 8. Taken together, these data show that in the setting of COVID-19, antigen-specific T cell responses correlate with the formation of SARS-CoV-2–specific antibodies and potentially help with increasing the overall antibody avidity.

## Discussion

In this study, we longitudinally investigate systemic cellular and humoral immunity from convalescent COVID-19 samples, comparing those with a prolonged symptom duration with those who experienced rapid symptom resolution. We evaluated a cohort of 50 convalesced patients, all of whom had samples collected at longitudinal time points: early convalescent phase (before 75 days following initial symptom onset), intermediate convalescent phase (76–150 days after symptom onset), and late convalescent phase (151 days or longer after symptom onset). In the studied cohort, 20 individuals had a prolonged duration of symptoms lasting 30 to 208 days after initial symptom onset. Although few differences between groups were observed based on immunophenotyping, we observed increases in antigen-specific CD4^+^ T cell responses to the SARS-CoV-2 S-protein in COVID-19 patients with prolonged symptom duration at the late convalescent time point. The prolonged group also exhibited increased antigen-specific activation in the cTfh and CD8^+^ T cell populations during the late phase. Individuals who recovered quickly from COVID-19 infection showed a gradual decrease in their T cell response magnitude over time, consistent with prior reports (16, 18). In contrast, individuals with prolonged symptoms had sustained T cell response magnitudes, with an increased overall response magnitude in late convalescence when compared with recovered individuals. Taken together, these data show a divergence in the T cell response magnitude over time between prolonged and recovered cohorts. To our knowledge, this is the first study to show an increased T cell response magnitude in individuals with prolonged symptom duration following COVID-19 infection. Consistent with the increased S-specific CD4^+^ T cell response, individuals with prolonged symptoms developed a higher avidity S-specific plasma antibody response, suggesting greater affinity maturation as compared with individuals who recovered quickly. Antibody avidity significantly correlated with S-specific CD4^+^ T cell responses in the later time points (Figure 6D), further suggesting that ongoing germinal center reactions may be occurring in individuals with prolonged symptoms.

Using flow cytometric assays, we assessed the frequencies of immune cell subsets, including T cells, B cells, NK cells ,and monocytes. Although we observed changes in immunologic signatures longitudinally, we observed no significant differences between prolonged and recovered groups. Using a variety of activation, exhaustion, memory, and senescence markers, we observed few differences between individuals with prolonged symptoms and those without. We detected a significant increase in the central memory (CD45RA^–^CCR7^+^) CD4^+^ T cell compartment for individuals in our prolonged group at all 3 time points (Supplemental Figure 3). This finding is potentially interesting as central memory T cells are known to be longer lived than effector T cells (48); this suggests a potential role in the increased CD4^+^ T cell response magnitude that was observed during late convalescence in prolonged individuals. To support this, similar observations were made in individuals recovering from SARS infection, with the CD4^+^ central memory population inducing a majority of the antigen-specific population (49). However, this observation in SARS-CoV-2 requires further investigation that includes other memory markers, such as CD27. No substantial differences were evident in the B cell compartment between individuals with prolonged symptoms and those without. Collectively, these data show that there are few immunophenotypic differences between individuals with prolonged symptom duration and those who recovered quickly, although these results should be verified in larger cohorts. Additional studies should evaluate lymphoid compartments beyond peripheral blood. For instance, these findings do not exclude shifts in T cell and monocyte populations that may be observed in bronchoalveolar lavage or lung tissue biopsy, particularly for those patients experiencing persistent respiratory symptoms.

These findings also identify differing antigen-specific immune signatures in both T cell and antibody compartments between individuals who have persistent COVID-19 symptoms as compared with those with resolved illness. Future studies should focus on determining the biological relevance of these maintained T cell responses. These observations lend support to the hypothesis that persistent viral reservoirs may contribute to the prolonged symptom duration in at least a subset of COVID-infected individuals. In the cohort studied here, nasal swabs collected during the early and intermediate phases of convalescence did not detect SARS-CoV-2 RNA; however, a lack of viral detection by nasal swab in the upper respiratory tract does not rule out the possible persistence of viral antigens in the body, particularly given the broad expression of angiotensin-converting enzyme 2, the entry receptor for SARS-CoV-2 (50–52). Persistent exposure of T cells to antigen could be an explanation for the maintained T cell response magnitudes observed in this study. If present, these antigens could sustain localized inflammatory responses and cause a prolonged symptom duration. Evidence for this hypothesis was demonstrated by SARS-CoV-2 protein and RNA being detected in various organs from autopsy patients, including lungs, kidney, intestines, and heart (53–57). Also, persistent SARS-CoV-2 has been detected in 50% of convalescent patients by rectal biopsy (22). Although these patients were asymptomatic at the time of biopsy, this demonstrates that SARS-CoV-2 can persist in tissue for long periods of time after resolution of acute infection. It has also been well described that prolonged viral shedding occurs in a subset of patients who no longer have symptoms but have nasopharyngeal swabs that remain PCR^+^ for weeks following initial symptom onset (58). Another study has shown that a small subset of these individuals with prolonged PCR^+^ test results exhibit increased CD8^+^ T cell responses (59); this further supports that persistent antigen exposure could be responsible for the maintained T cell response.

An additional implication of our study is that patients with prolonged symptoms do not exhibit an impairment in longitudinal SARS-CoV-2–specific T cell memory formation. This is based on the fact that prolonged individuals had similar antigen-specific T cell responses at the early and intermediate phases of convalescence and even had increased response at the late time point. Other studies have shown that recovered individuals exhibit antigen-specific cytokine production, even in asymptomatic individuals (17, 60). Future projects should focus on investigating functional differences between prolonged and recovered groups. Despite the increased T cell response magnitudes observed in individuals from the prolonged group, it is possible that their T cell responses may be less functional, therefore allowing a persistence of SARS-CoV-2 virus. This was previously observed in a single individual with prolonged symptoms, persistent PCR^+^ test results, and an impaired IFN-γ T cell response (61). This scenario, along with our findings, indicates that COVID-19 vaccination may provide therapeutic benefits to a subset of patients with PASC (62). However, studies investigating greater numbers are warranted.

Our study has a few limitations. Patients with PASC vary in presentation and encompass heterogenous syndromes; future studies with larger cohorts should investigate whether certain clinical manifestations are associated with maintained SARS-CoV-2–specific immune responses. In our cohort, individuals with prolonged symptoms were more likely to require hospitalization as compared with the recovered group. This is not unexpected given prior reports that have shown higher frequencies of prolonged symptom duration in those individuals who have more severe infection (37). We attempted to control for this variability by running a linear mixed effects model comparing the differences between hospitalized samples, prolonged nonhospitalized samples, and recovered nonhospitalized samples. Our results showed little evidence of significant differences between hospitalized and nonhospitalized individuals within the prolonged group, but these results should be investigated further in a larger cohort. As stated previously, we found no differences based on immune cell phenotype in these samples, but this observation is restricted to systemic responses observed in peripheral blood. Although we would expect major differences to be detectable in the peripheral blood, further investigation into tissues where inflammation may be causing immune dysregulation and persistent symptoms is needed.

In conclusion, our study provides a thorough investigation into the immune response of COVID-19 patients with prolonged symptom duration. While we observed few differences in nonspecific immune activation between groups, individuals with prolonged symptoms were found to have increased SARS-CoV-2–specific immune responses during late convalescence. These results have many important implications, emphasizing the need for future efforts to verify and expand upon these data.

## Methods

### Sample collection.

Peripheral blood samples were collected as previously described (1). In short, peripheral blood samples were collected from convalescent, previously COVID-infected individuals (*n* = 50). Samples from all individuals were collected at multiple time points. A majority of patients had samples collected for all 3 time points (*n* = 38), while the remainder had samples for at least 2 time points (*n* = 12). All samples were collected prior to the participants receiving any COVID-19 vaccines. Patient data for hospitalized individuals, such as medications, were collected from University of Alabama at Birmingham’s (UAB) electronic medical record, and patient-reported clinical data were collected for all patients and uploaded to REDCap at the time of initial sample collection (63). All data and sample collection were done in accordance with UAB’s IRB. All patients had a confirmed positive SARS-CoV-2 PCR nasal swab or were positive for N-protein–specific antibodies. Patient data were utilized to determine peak ordinal scores that have been described by other clinical studies (64). In brief, patients exhibited scores of 2 (symptomatic but no hospitalization), 4 (hospitalization requiring medical care but no oxygen), 5 (hospitalization requiring both medical care and oxygen), and 7 (hospitalization requiring invasive mechanical ventilation). An overview of the cohort clinical data is given in Table 1, with more specific data on each individual patient shown in Supplemental Table 1. In addition, there were samples from 10 healthy individuals (CoV^–^) collected between 2014 and 2018.

### Flow cytometric phenotypic analyses.

Isolated PBMCs were thawed in R10 media, RPMI (Gibco, Thermo Fisher Scientific) with 10% human AB serum (Gemini), and then stained using 1 of 3 different phenotyping panels. Immune cell subsets and general T cell activation/senescence were identified with the following panel: CD45-Pecy7 (BD Biosciences; clone HI30), CD16-FITC (Invitrogen, Thermo Fisher Scientific; clone CD16), CD14-A700 (BD Biosciences; clone M5E2), CD19-BUV563 (BD Biosciences; clone SJ25C1), CD56-BV421 (BD Biosciences; clone NCAM16.2), CD3-A780 (Invitrogen, Thermo Fisher Scientific; clone UCHT1), CD8-V500 (BD Biosciences; clone RPA-T8), CD4-BV785 (BD Biosciences; clone SK3), HLADR-PE (BD Biosciences; clone G46-6), CD38-BUV737 (BD Biosciences; clone HB7), CD28-APC [BD Biosciences; clone CD28.2(RUO)], and CD57-Percpcy55 (BioLegend; clone HNK-1). T cells were further analyzed using the following panel: CD3-A780 (Invitrogen, Thermo Fisher Scientific; clone UCHT1), CD4-PE Alexa Fluor 610 (Invitrogen, Thermo Fisher Scientific; clone RPA-T4), CD8-FITC (BD Biosciences; clone SK1), CD14-BUV563 (BD Biosciences; clone MφP9), CD19-BUV563 (BD Biosciences; clone SJ25C1), CD69-BUV737 (BD Biosciences; clone FN50), OX40-Pecy7 (BioLegend; clone Ber-ACT35), CD154-APC (BD Biosciences; clone TRAP1), CD137-BV650 (BD Biosciences; clone 4B4-1), PD1-BV785 (BioLegend; clone EH12.2H7), TIGIT-A700 (R&D Systems, Bio-Techne; clone 741182), TIM3-BV421 (BioLegend; clone F38-2E2), PDL1-PE (BD Biosciences; clone MIH1), CD45RA-BV510 (BD Biosciences; clone HI100), and CCR7-Percpcy5.5 (BD Biosciences; clone 150503). B cell phenotype was assessed using the following panel: IgD-FITC (BD Biosciences; clone IA6-2), CD4-BB790 (BD Biosciences; clone L200), CD38-AF647 (Santa Cruz Biotechnology; clone AT1), CD19-AF700 (Beckman Coulter; clone A78837), CD20-APC-Cy7 (BD Biosciences; clone L27), CD11c-BV421 (BD Biosciences; clone 3.9), CD27-BV650 (BioLegend; clone O323), CD69-BUV395 (BD Biosciences; clone FN50), CD8-BUV496 (BD Biosciences; clone RPA-T8), CD95-BUV737 (BD Biosciences; clone DX2), CD21-PECy5 (BD Biosciences; clone B-Ly4), and CD138-PECy7 (Bio-Legend; clone DL-101). All panels identified dead cells by utilizing Live/Dead Blue Stain (Life Technologies, Thermo Fisher Scientific). Samples were fixed with a 1% formalin solution. Analysis of samples was performed using FACS Symphony A3 (BD Biosciences) flow cytometer within 24 hours of staining and analyzed using FlowJo (v10) software. Representative gating strategies for all 3 phenotyping panels are shown in Supplemental Figures 9–11.

UMAP plot formation was performed in FlowJo using previously designed plug-ins. In short, 1000 cells from each sample were concatenated into a single flow cytometry file. The Phenograph 3.0 plug-in (65) was used to identify clusters for all 3 UMAP algorithms. The UMAP 3.1 plug-in was then utilized to make all 3 UMAP plots. Settings for UMAP generation included Euclidean distance, nearest neighbors set to 15, minimum distance set to 0.5, and 2 total components.

### Antigen-specific AIM assay.

Samples underwent peptide stimulation to assess upregulation of activation-induced markers as described by other groups (44, 45). In brief, after PBMCs were thawed and rested for 3 hours, samples were stimulated for 18 hours using pooled SARS-CoV-2 S-protein peptides (BEI Resources). This pool included 181 peptides (primarily 17-mers with 10–amino acid overlap) spanning the S-glycoprotein of the USA-WA1/2020 strain of SARS-CoV-2. Stimulation occurred at an individual peptide concentration of 1 μg/mL, with the unstimulated condition receiving an equimolar concentration of DMSO. Following stimulation, cells were stained with the following panel: CD3-A780 (Invitrogen, Thermo Fisher Scientific; clone UCHT1), CD4-BUV563 (BD Biosciences; clone SK3), CD8-FITC (BD Biosciences; clone SK1), CD14-A700 (BD Biosciences; clone M5E2), CD19-A700 (BD Biosciences; clone HIB19), OX40-Pecy7 (BioLegend; clone Ber-ACT35), PDL1-PE (BD Biosciences; clone MIH1), CD69-BUV737, CD137-BV650 (BD Biosciences; clone 4B4-1), PD1-BV785 (BioLegend; clone EH12.2H7), and CXCR5-BV421 (BD Biosciences; clone RF582). Samples were then fixed with a 1% formalin solution and analyzed on a Symphony A3 (BD Biosciences) flow cytometer within 24 hours of staining. Analysis was completed using FlowJo (v10) software, and a representative gating strategy is shown in Supplemental Figure 12.

Overall antigen-specific activation was primarily determined by looking at the CD4 response magnitude and coexpression of OX40 and PD-L1. These markers were chosen after previous studies found the antigen-induced expression of these markers on CD4^+^ T cells and T follicular helper cells across several rhesus macaque and human studies (44, 45, 66). Unstimulated and stimulated examples of CD4^+^ T cells are shown at all 3 time points for 4 individuals in Figure 3A. cTfh activation was determined by investigating coexpression of OX40 and PD-L1. Finally, CD8 activation was determined by dual expression of CD69 and CD137, as shown by other groups (16, 18). Net T cell response magnitudes were calculated by subtracting the expression following SARS-CoV-2 stimulation from the unstimulated condition. The positivity criteria threshold was set to those responses that showed an upregulation of the given activation markers at least 3 times higher than unstimulated negative controls and had a Fisher’s exact *P* value less than or equal to 0.0001; these positivity criteria were adapted from previous flow cytometric optimization assays (67) and have been used by our group in previous projects involving antigen-specific flow cytometric assays (68).

### Antibody ELISA.

SARS-CoV-2 ELISAs were performed as previously described (14, 21). Briefly, 96-well plates were coated with 0.5 μg/mL of recombinant SARS-CoV S1 and S2 (catalog 40589-V08B1, SinoBiological Inc) or recombinant SARS-CoV-2 N (catalog 40588-V08B, SinoBiological Inc) overnight at 4°C. The next day, plates were blocked with 3% BSA in PBS for 1 hour at room temperature. Plates were washed and the sera diluted at 1:1000 in PBS containing 0.05% Tween-20 (PBST) and added to the plates in duplicates before being incubated for 1 hour at room temperature. Plates were washed and 8 M urea (catalog ZU10001, Invitrogen, Thermo Fisher Scientific) or PBST only was added for 15 minutes. Plates were washed and anti-human IgG-HRP (catalog 109-035-008, Jackson ImmunoResearch) at 1:2000 dilution was added and developed by KPL SureBlue (catalog 5120-0077, Seracare) TMB Substrate. Readouts were recorded as the OD at 450 nm. Avidity index was calculated as follows: (normalized OD of urea-treated sera/normalized OD urea-untreated sera) × 100.

### Statistics.

All statistical analyses and figure generation were performed using R. Significance was determined using either a Wilcoxon rank sum test for unpaired analyses between prolonged and recovered groups or a Wilcoxon signed-rank test for paired analyses between longitudinal samples. Relationships between antigen-specific net frequencies of activation markers and DPSO were modeled using a linear mixed effects model with random intercept to account for repeated measures (model graphs shown in Figure 3B, Figure 4B, and Supplemental Figure 6B). Additionally, linear mixed effects modeling was used to assess if differences between prolonged and recovered groups were not driven by hospitalization status. *P* < 0.05 was considered significant. Our findings did not show differences in CD4 response magnitude between hospitalized and nonhospitalized prolonged individuals (data not shown); more investigation should be done with increased numbers. Three individuals had potentially outlying observations based on T cell number and phenotype. Sensitivity analyses were performed without these participants; 1 individual with low T cell numbers was excluded from all T cell analyses, while no differences in T cell results were observed for the remaining 2 individuals, validating their inclusion in the data set.

### Study approval.

All data and sample collection from subjects who gave written informed consent were done in accordance with and with the approval of UAB’s IRB.

## Author contributions

JKF identified the cohort, designed/performed the cell subset and T cell flow cytometric assays, designed/performed all antigen-specific T cell assays, analyzed all corresponding data, generated all figures, and wrote the initial draft of the manuscript. S Sarkar performed/analyzed all B cell phenotyping assays and antibody binding/affinity assays. TRF assisted with staining of T cell phenotyping assays. SB assisted with initial R coding files. S Sterrett assisted with collection and organization of all biological samples. KQ and AB assisted with initial experimental design. DL assisted with all biostatistical analyses. S Sabbaj, JJK, PAG, and NE provided all reagents for assays and conceptualized/designed the study. JJK and S Sarkar provided the initial written draft pertaining to B cell and antibody data. NE provided feedback in regard to experimental design and comments on the initial manuscript. S Sarkar, TRF, AB, DML, S Sabbaj, JJK, PAG, and NE provided final edits on the manuscript.

## Supplementary Material

Supplemental data

## Figures and Tables

**Table 1:**
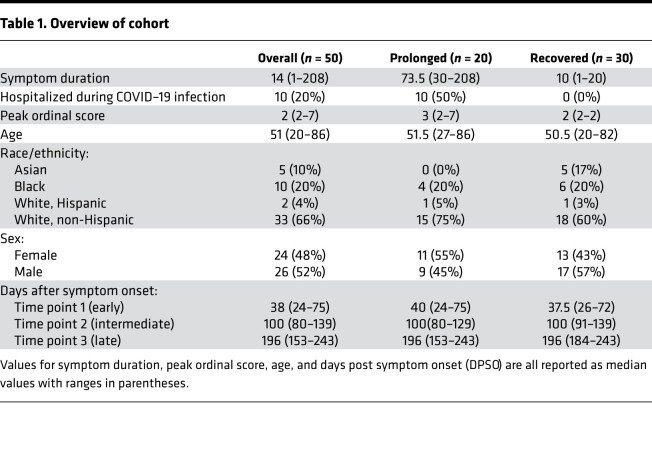
Overview of cohort

## Version 1 06/18/2021

In-Press Preview

## Version 2 08/09/2021

Electronic publication
