# Supplementary material for: Duration of post–COVID-19 symptoms is associated with sustained SARS-CoV-2–specific immune responses
Source: JCI Insight. 2021 Aug 9;6(15):e151544. doi: 10.1172/jci.insight.151544 (PMC8410022; doi:10.1172/jci.insight.151544)
Supplement: Supplemental data [file jciinsight-6-151544-s174.pdf]

PTID	Race	Ethnicity	Sex	PCR+	Ab+	Hospitalization Status	Peak Ordinal Score	Symptom Presentation								Symptom Duration	Medications In Acute Infection	Comobidities
								Dyspnea	Fatigue	Psychataxia	Cough	Fever	Myalgia	GI Upset	Anosmia/Aguesia			
P01	C	H	F	Y	Y	Y	5	X	X	X	X	X	X	X		148		obesity
P02	C	NH	F	Y	N	Y	4	X	X	X	X	X	X	X	X	136	Steroids	obesity, asthma
P03	C	NH	M	n/a	Y	N	2		X	X	X	X	X		X	47		
P04	C	NH	F	Y	N	N	2	X	X	X	X	X	X	X		208		
P05	C	NH	F	Y	Y	N	2	X	X		X	X		X	X	40		sleep apnea, Afib
P06	C	n/a	F	Y	N	N	2		X		X				X	42		
P07	C	NH	F	Y	Y	N	2	X	X	X	X	X	X	X	X	33		
P08	C	NH	F	Y	Y	N	2	X	X		X	X	X	X	X	76		
P09	AA	NH	M	Y	Y	N	2	X			X	X	X	X	X	94		
P10	C	NH	M	N	Y	Y	n/a		X	X	X	X		X	X	188	n/a	
P11	AA	NH	F	Y	Y	Y	5	X		X	X	X		X	X	198		diabetes, HTN
P12	C	NH	M	Y	Y	Y	5	X	X		X		X	X	X	200		HTN, bypass surgery
P13	C	NH	F	Y	Y	Y	5	X	X	X	X	X	X		X	90	Remdesivir	HTN, diabetes, sleep apnea
P14	C	NH	M	Y	N	N	2	X								62		asthma
P15	C	NH	F	Y	Y	N	2	X	X	X	X	X	X		X	99		SVT
P16	C	NH	F	Y	Y	Y	n/a	X	X	X	X	X		X	X	115		pericarditis
P17	C	NH	M	Y	N	Y	5	X	X	X	X	X	X	X	X	45		HTN
P18	AA	NH	M	Y	Y	Y	5	X	X	X	X	X			X	30		HTN, diabetes
P19	C	NH	M	N	Y	N	2		X		X		X			34		
P20	AA	NH	M	Y	Y	Y	7	X	X	X	X	X	X	X	X	139	Remdesivir	diabetes, HTN, sleep apnea
R01	C	NH	M	Y	Y	N	2		X		X	X				3		HTN, Afib
R02	C	NH	F	Y	N	N	2				X					4		HTN
R03	C	NH	M	Y	Y	N	2	X					X			3		
R04	A	NH	F	Y	Y	N	2		X		X	X	X		X	16		
R05	A	NH	M	Y	Y	N	2		X	X	X	X	X	X	X	12		
R06	C	H	F	Y	Y	N	2	X			X	X	X	X	X	15		asthma
R07	C	NH	F	Y	N	N	2	X	X	X		X	X			12		
R08	C	NH	M	Y	N	N	2	X	X	X	X	X	X	X	X	12		
R09	C	NH	M	Y	Y	N	2		X			X	X			1		
R10	C	NH	M	Y	Y	N	2		X	X	X	X	X		X	8		sleep apnea
R11	C	NH	M	Y	Y	N	2		X	X	X	X	X	X		7		HTN
R12	C	NH	M	Y	Y	N	2	X	X		X	X		X		4		
R13	C	NH	M	Y	Y	N	2		X		X		X			10		Afib
R14	C	NH	F	Y	N	N	2	X	X	X	X		X	X		11		asthma, HTN
R15	C	n/a	F	Y	Y	N	2	X	X	X		X	X		X	14		
R16	AA	NH	F	Y	Y	N	2	X	X	X	X	X	X	X		16		
R17	A	NH	F	Y	N	N	2	X	X		X					15		
R18	C	NH	M	Y	Y	N	2		X	X	X	X	X	X		5		HTN
R19	C	NH	F	Y	N	N	2		X				X		X	7		
R20	AA	NH	F	Y	Y	N	2		X	X	X	X	X	X		20		
R21	AA	NH	M	n/a	Y	N	2	X	X	X	X	X	X	X	X	14		HTN
R22	C	NH	M	Y	Y	N	2		X					X		4		
R23	A	NH	M	Y	Y	N	2		X				X	X	X	5		
R24	C	NH	M	Y	Y	N	2		X	X	X	X	X		X	11		HTN
R25	A	NH	F	Y	Y	N	2		X		X	X	X		X	2		
R26	AA	NH	M	Y	Y	N	2	X	X			X	X		X	3		
R27	C	NH	M	Y	N	N	2		X		X		X		X	7		
R28	AA	NH	F	Y	Y	N	2		X		X	X	X		X	5		
R29	AA	NH	F	Y	Y	N	2				X	X	X	X		8		
R30	C	NH	M	n/a	Y	N	2	X	X	X	X	X				14		HTN

Supplemental Table 1: Individual Patient Data. PTID: patient ID; P#=prolonged group, R#=recovered group. Race: C=Caucasian, AA=African American, A= Asian. Ethnicity: H=Hispanic, NH=non-Hispanic. PCR+: result of RT-PCR SARS-CoV-2 diagnostic test. Ab+: result of N-protein-specific antibody test. Peak ordinal score: score from 2-7 based on WHO ordinal scale. Medications in acute infection: medications used during treatment of acute COVID infection during hospitalization.

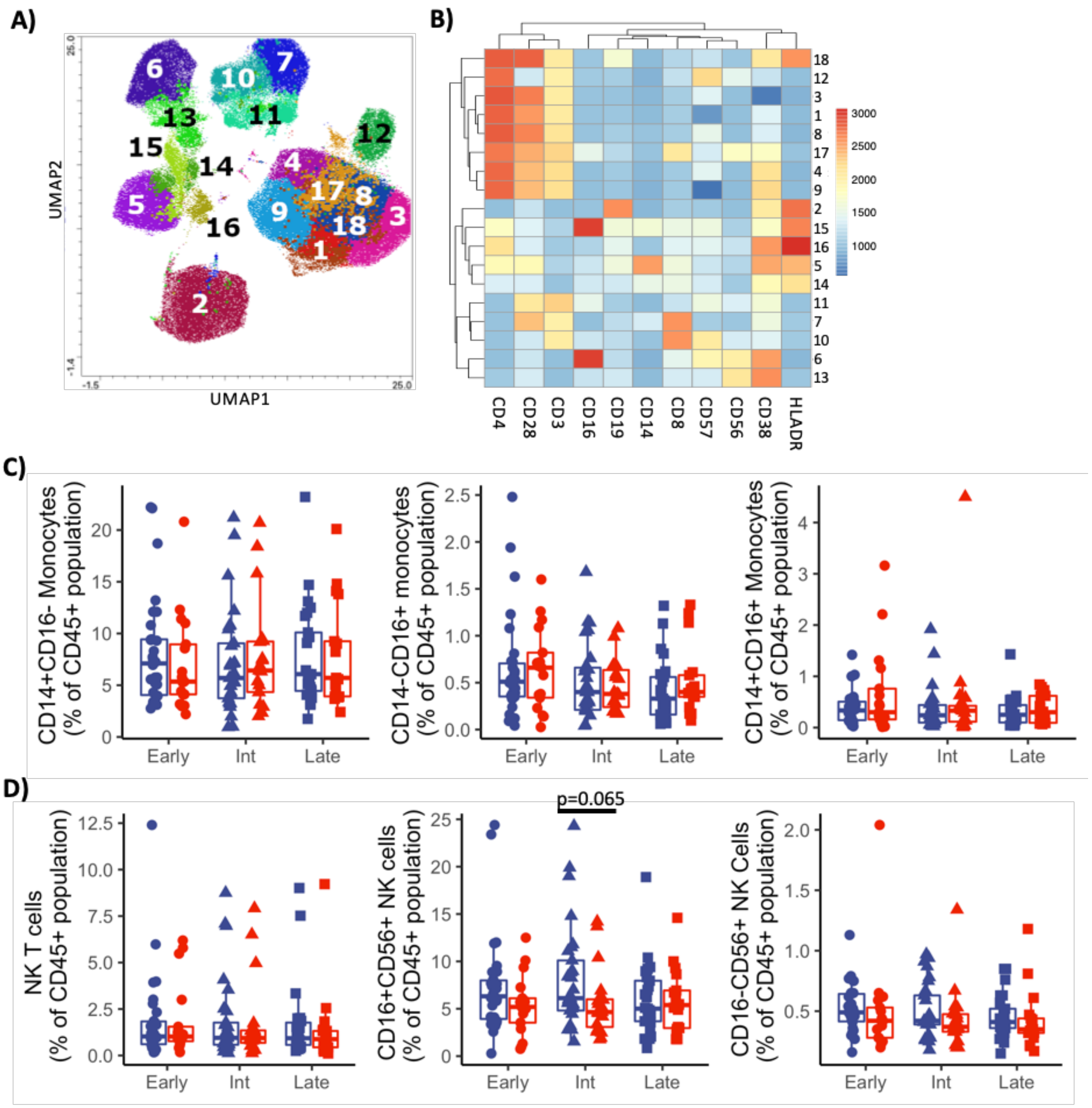

Supplemental Figure 1: Frequencies of innate immune cells show no difference between prolonged and recovered groups. Investigation into recovered (n=30) and prolonged (n=20) groups and the frequencies of innate immune cell subsets including: **A-B)** Overview of UMAP clustering and corresponding heatmap with 18 total clusters identified. **C)** CD14+, CD16+, and CD14+CD16+ monocyte populations; **D)** NK T cells, CD16+CD56+ NK cells and CD16-CD56+ NK cells. Navy=recovered group, red=prolonged group; circles=early, triangles=intermediate, squares=late. Boxplots indicate median, IQR, and 95% confidence interval; significance determined by the unpaired Wilcoxon rank sum tests.

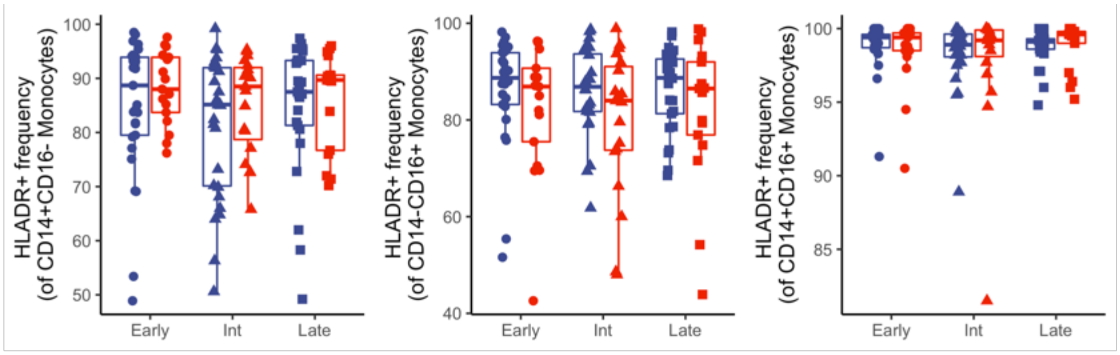

Supplemental Figure 2: HLA-DR expression shows no difference between prolonged and recovered groups. Investigation into HLA-DR expression on different monocyte subsets in recovered (n=30) and prolonged (n=20) individuals. HLA-DR expression is shown on CD14+ (left), CD16+ (middle), and CD14+CD16+ (right) monocyte populations. Navy=recovered group, red=prolonged group; circles=early, triangles=intermediate, squares=late. Boxplots indicate median, IQR, and 95% confidence interval; no significance determined by the unpaired Wilcoxon rank sum tests.

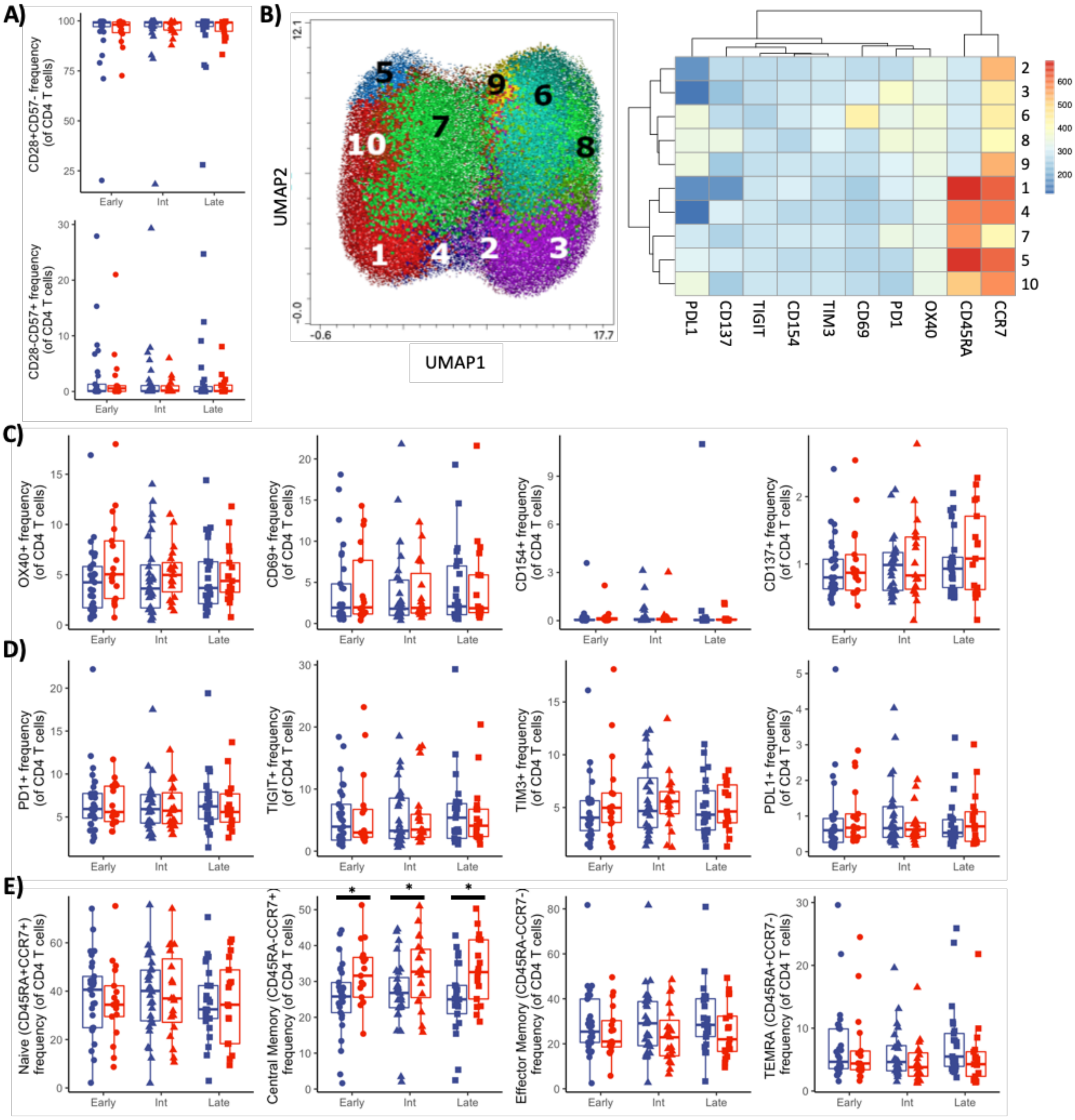

Supplemental Figure 3: CD4+ T-cell phenotyping findings show few differences between prolonged and recovered groups. A) Frequency of activated memory (CD28+CD57-) and senescent (CD28-CD57+) populations. **B)** Overview of UMAP clustering and corresponding heatmap with 10 total clusters identified. **C)** Expression of activation markers: OX40, CD69, CD154, and CD137. **D)** Expression of exhaustion markers: PD1, PDL1, TIGIT, and TIM3. **E)** Frequency of effector/memory populations: Naive (CD45RA+CCR7+), central memory (CD45RA-CCR7+), effector memory (CD45RA-CCR7-), and TEMRA (CD45RA+CCR7-). Navy=recovered group, red=prolonged group; circles=early, triangles=intermediate, squares=late. Boxplots indicate median, IQR, and 95% confidence interval; significance determined between recovered (n=29) and prolonged (n=20) groups by the unpaired Wilcoxon rank sum tests are indicated as follows: *P ≤ 0.05, **P ≤ 0.01, ***P ≤ 0.001.

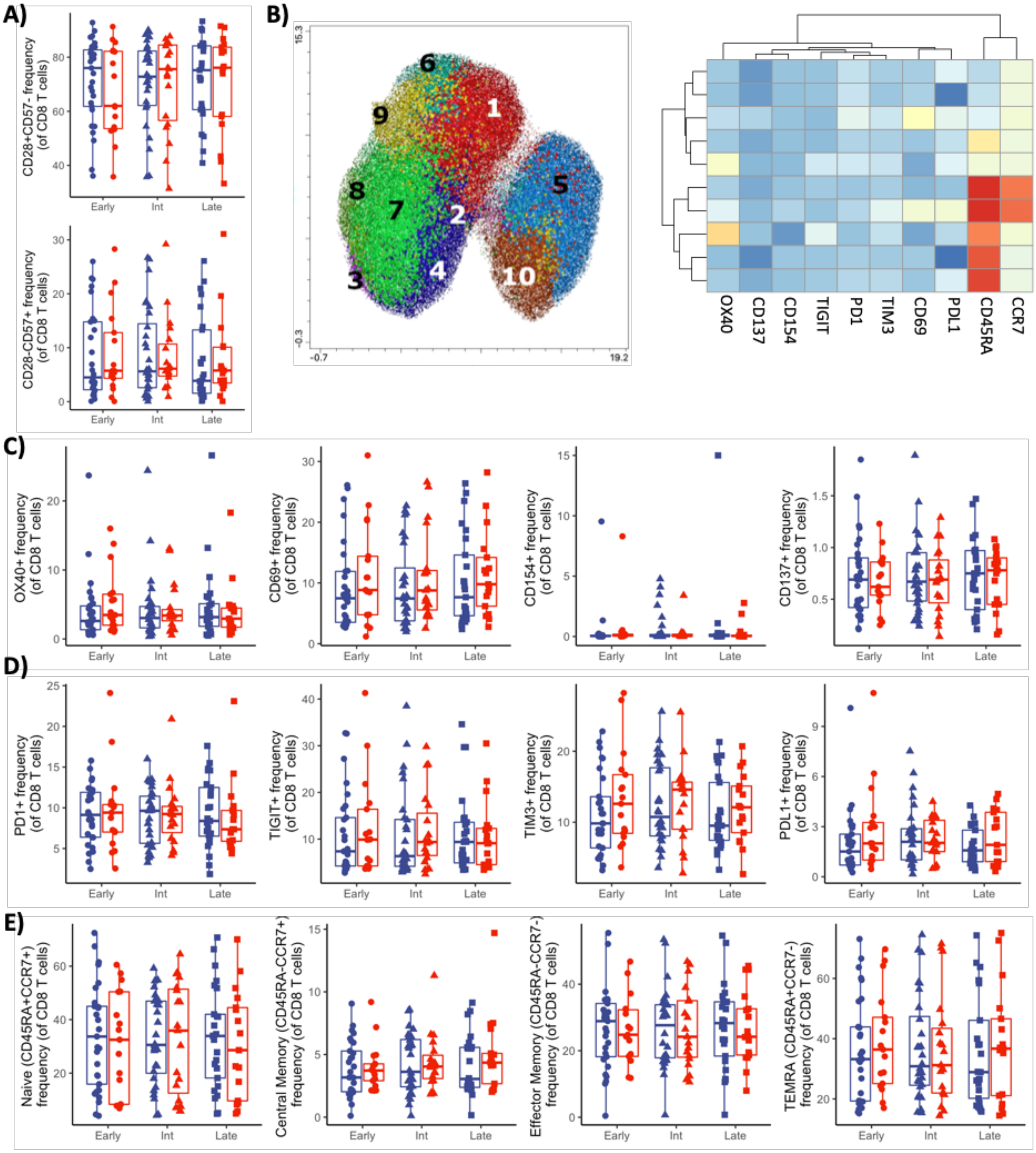

Supplemental Figure 4: CD8+ T-cell phenotyping findings show no difference between prolonged and recovered groups. A) Frequency of activated memory (CD28+CD57-) and senescent (CD28-CD57+) populations. **B)** Overview of UMAP clustering and corresponding heatmap with 10 total clusters identified. **C)** Expression of activation markers: OX40, CD69, CD154, and CD137. **D)** Expression of exhaustion markers: PD1, PDL1, TIGIT, and TIM3. **E)** Frequency of effector/memory populations: Naive (CD45RA+CCR7+), central memory (CD45RA-CCR7+), effector memory (CD45RA-CCR7-), and TEMRA (CD45RA+CCR7-). Navy=recovered group, red=prolonged group; circles=early, triangles=intermediate, squares=late. Boxplots indicate median, IQR, and 95% confidence interval; no significance determined between recovered (n=29) and prolonged (n=20) groups by the unpaired Wilcoxon rank sum tests.

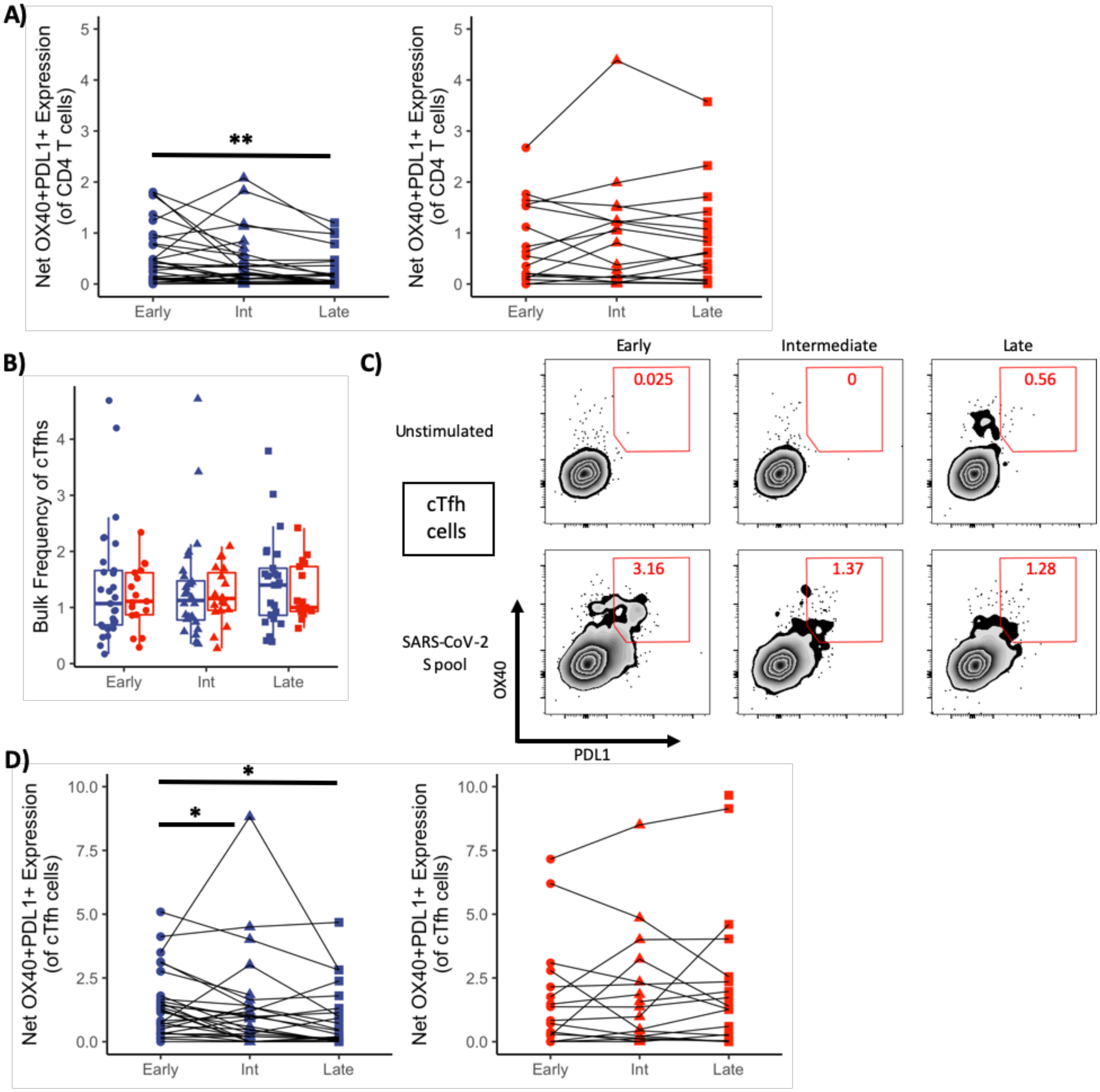

Supplemental Figure 5: Investigation into activation marker expression in CD4+ and cTfh T-cell subsets. **A)** Comparisons of the net OX40+PDL1+ response magnitude in the CD4+ T cell subset between recovered (n=29) and prolonged (n=20) groups in a paired manner; significance between groups determined by paired Wilcoxon signed rank test. **B)** Overall frequencies of cTfh cells (CXCR5+PD1+) as a percentage of the overall CD4+ T-cell population in recovered (n=29) and prolonged (n=20) groups; no significance determined by the unpaired Wilcoxon rank sum tests. **C)** Representative example of cTfh activation marker gating strategy. **D)** Comparisons of the net OX40+PDL1+ response magnitude in the cTfh T cell subset between recovered (n=29) and prolonged (n=20) groups in a paired manner; significance between groups determined by paired Wilcoxon signed rank test. Navy=recovered group, red=prolonged group; circles=early, triangles=intermediate, squares=late. Boxplots indicate median, IQR, and 95% confidence interval; signficance indicated as follows: *P ≤ 0.05, **P ≤ 0.01, ***P ≤ 0.001.

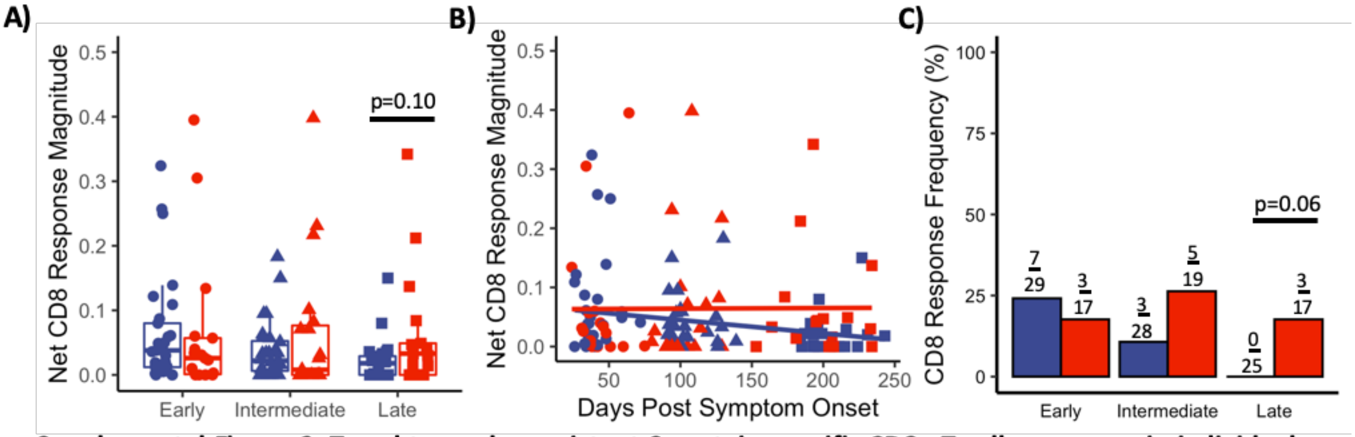

Supplemental Figure 6: Trend towards persistent S-protein-specific CD8+ T-cell responses in individuals with prolonged symptom duration. Investigation into the upregulation of activation markers CD69 and CD137 in CD8+ T-cells. **A)** Comparisons of the net CD69+CD137+ response magnitude between recovered (n=29) and prolonged (n=20) groups; significance between groups determined by unpaired Wilcoxon rank sum. **B)** Comparisons of the net CD8+ T-cell response magnitude longitudinally by days post symptom onset (n=135 across all three timepoints); significance determined by linear mixed effect modeling. **C)** Frequencies of CD8+ T-cell responses meeting positivity criteria; significance determined by Fisher's exact test. Navy=recovered group, red=prolonged group; circles=early, triangles=intermediate, squares=late. Boxplots indicate median, IQR, and 95% confidence interval; significance is indicated as follows: *P ≤ 0.05, **P ≤ 0.01, ***P ≤ 0.001.

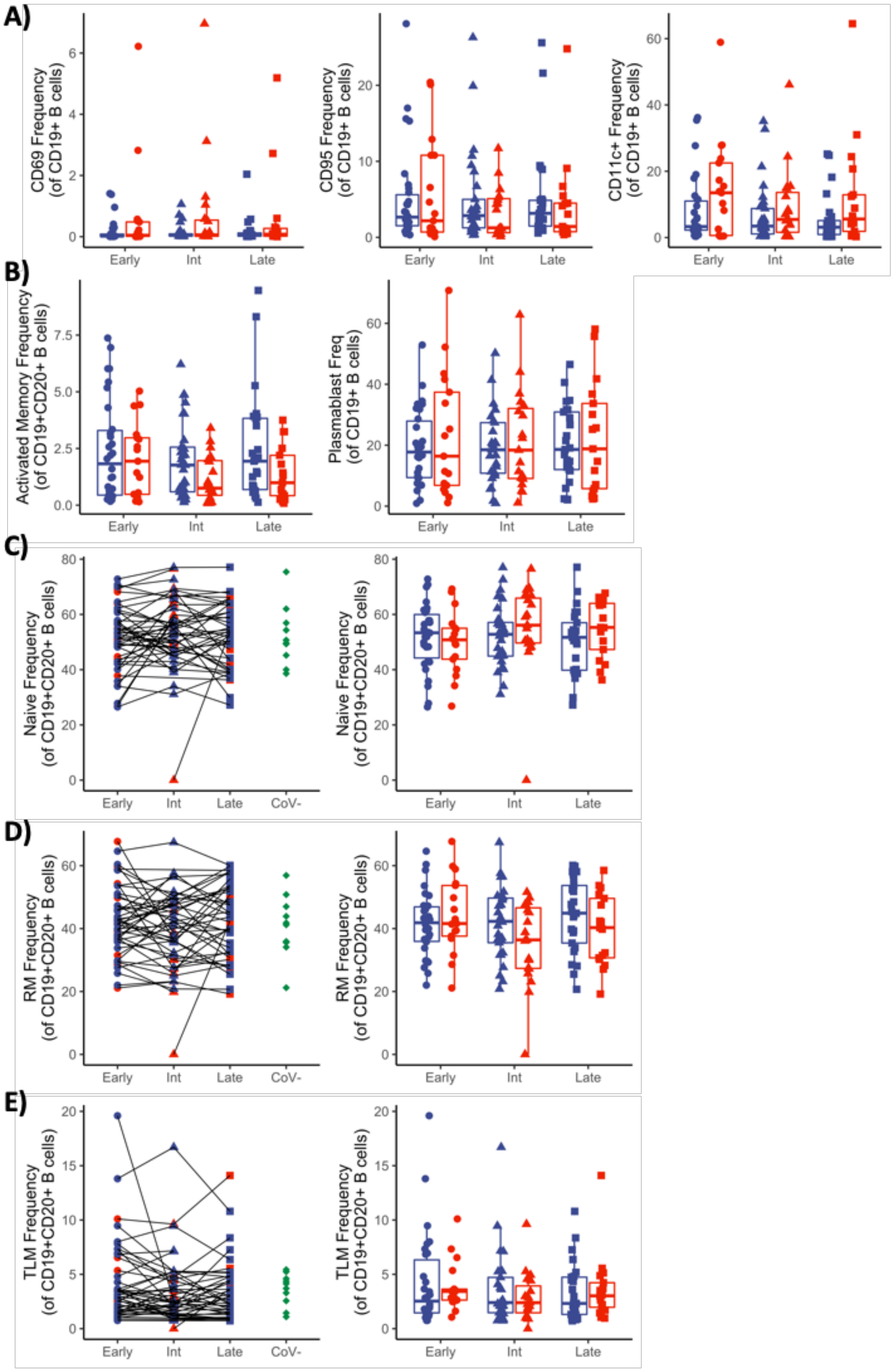

Supplemental Figure 7: B cell phenotyping data shows no differences between prolonged and recovered groups. A-B) Comparison between recovered (n=30) and prolonged (n=20) groups of the activation markers CD69, CD95 and CD11c, as well as the activated memory and plasmablast B-cell subsets. **C-E)** Longitudinal analysis and group comparison between recovered (n=30) and prolonged (n=20) groups of additional B-cell subsets: (C) Naïve B cell , (D) Resting Memory (RM) and (E) Tissue-like Memory (TLM). Navy=recovered group, red=prolonged group; circles=early, triangles=intermediate, squares=late. Boxplots indicate median, IQR, and 95% confidence interval; no significance between groups determined by unpaired Wilcoxon rank sum or paired Wilcoxon signed rank tests.

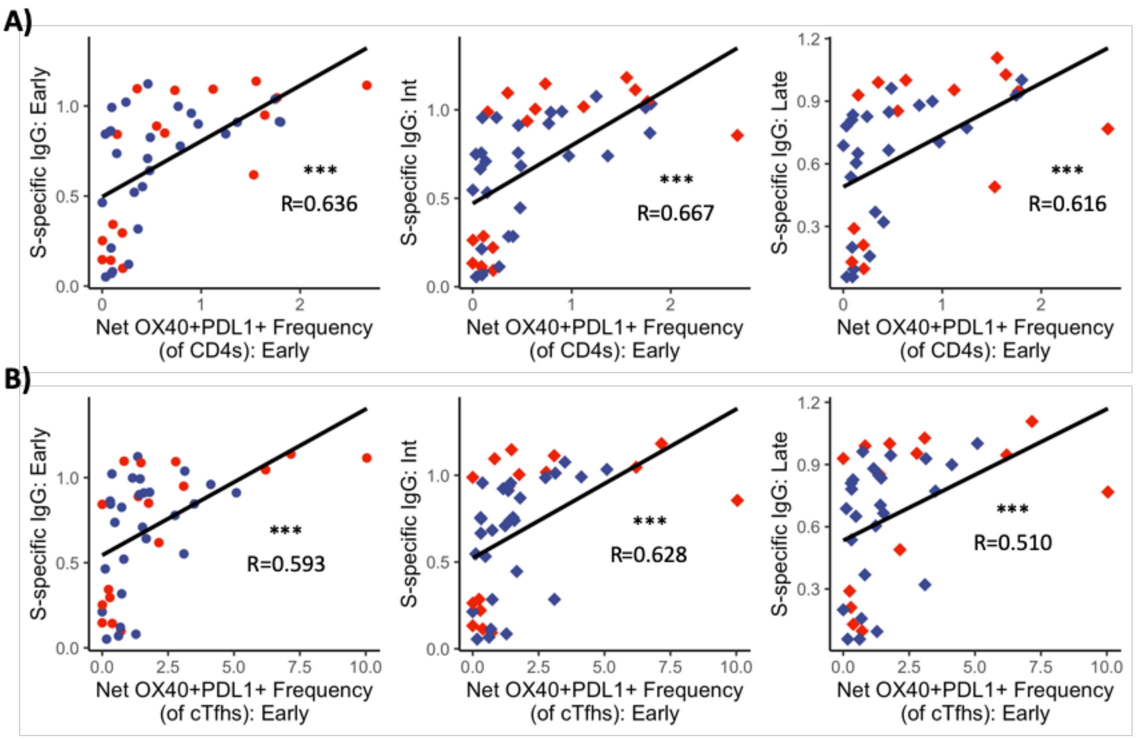

Supplemental Figure 8: Significant correlations between S-specific IgG with net CD4+ T-cell and cTfh response magnitudes. Investigation into correlations between antigen-specific T-cell responses and S-protein-specific IgG binding for all individuals (n=49). **A)** Correlation graphs between S protein-specific CD4+ T-cell response magnitude at the early timepoint and S-specific IgG at all three timepoints. **B)** Correlation graphs between S protein-specific cTfh T-cell response magnitude at the early timepoint and S-specific IgG at all three timepoints. Navy=recovered group, red=prolonged group; circles=early timepoint, diamonds=mismatch timepoint. Significance determined by Spearman's rank-order correlation and are denoted as follows: *P ≤ 0.05, **P ≤ 0.01, ***P ≤ 0.001.

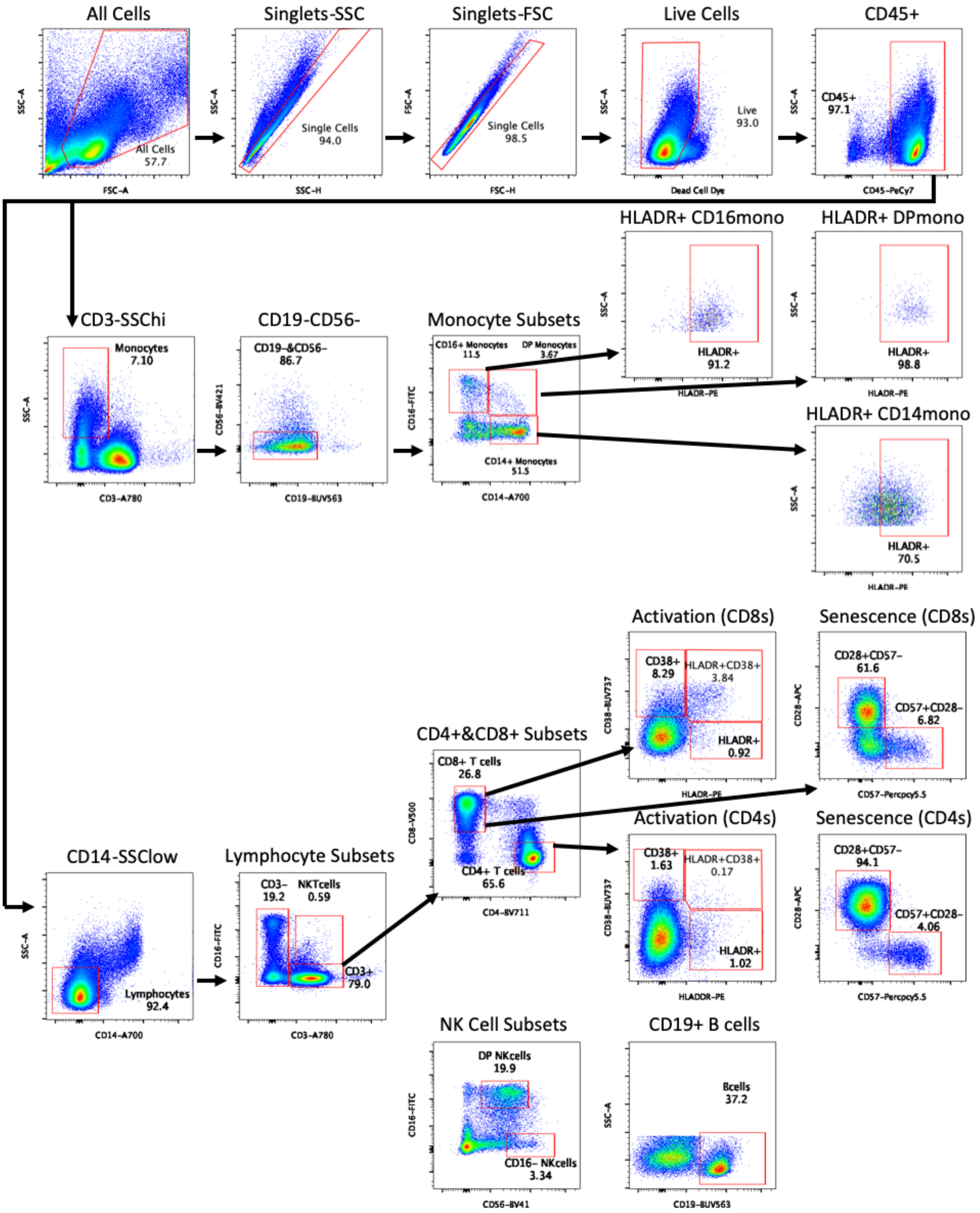

Supplemental Figure 9: Cell Subset Gating Strategy

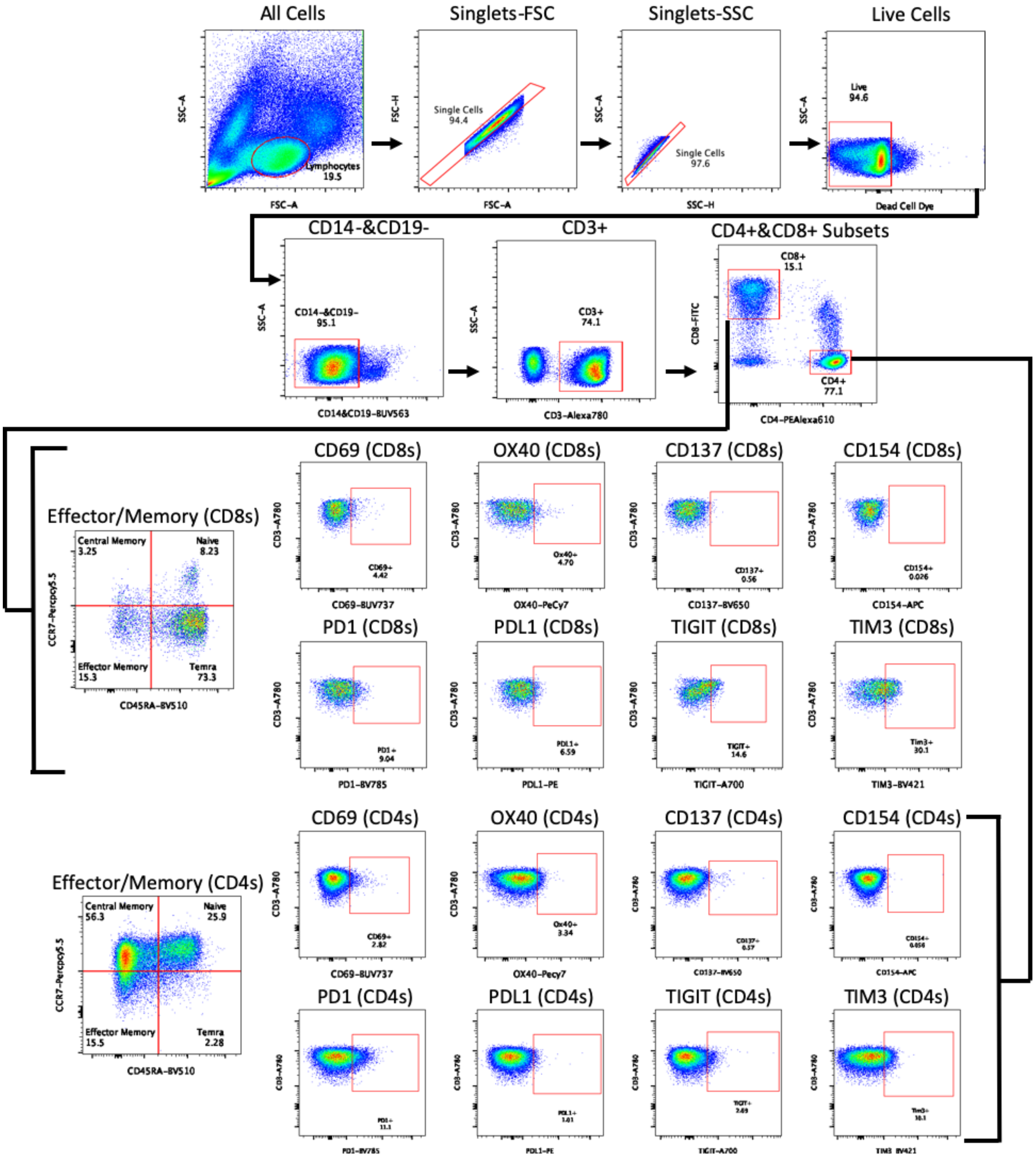

Supplemental Figure 10: T-cell Phenotyping Gating Strategy

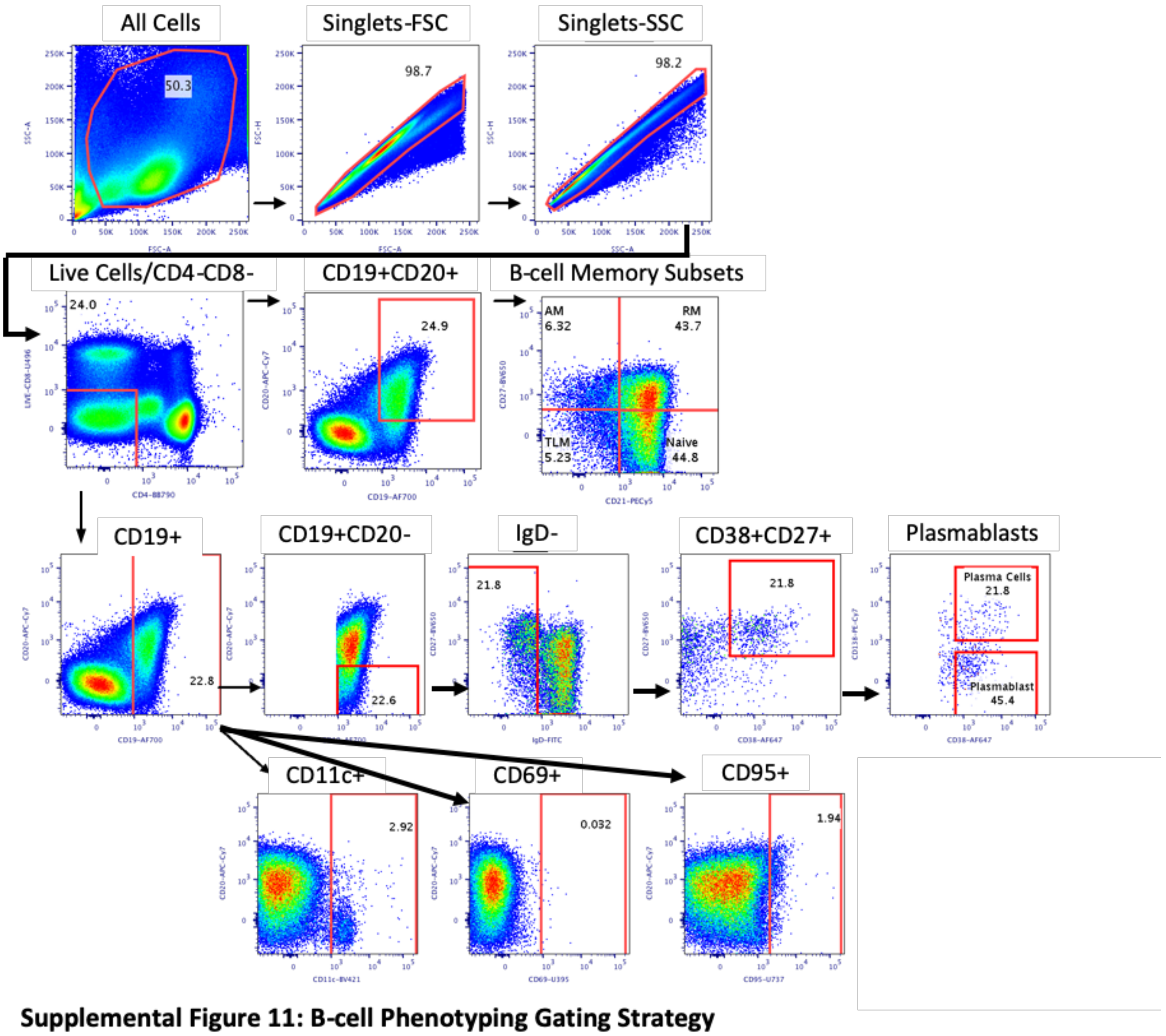

Supplemental Figure 11: B-cell Phenotyping Gating Strategy

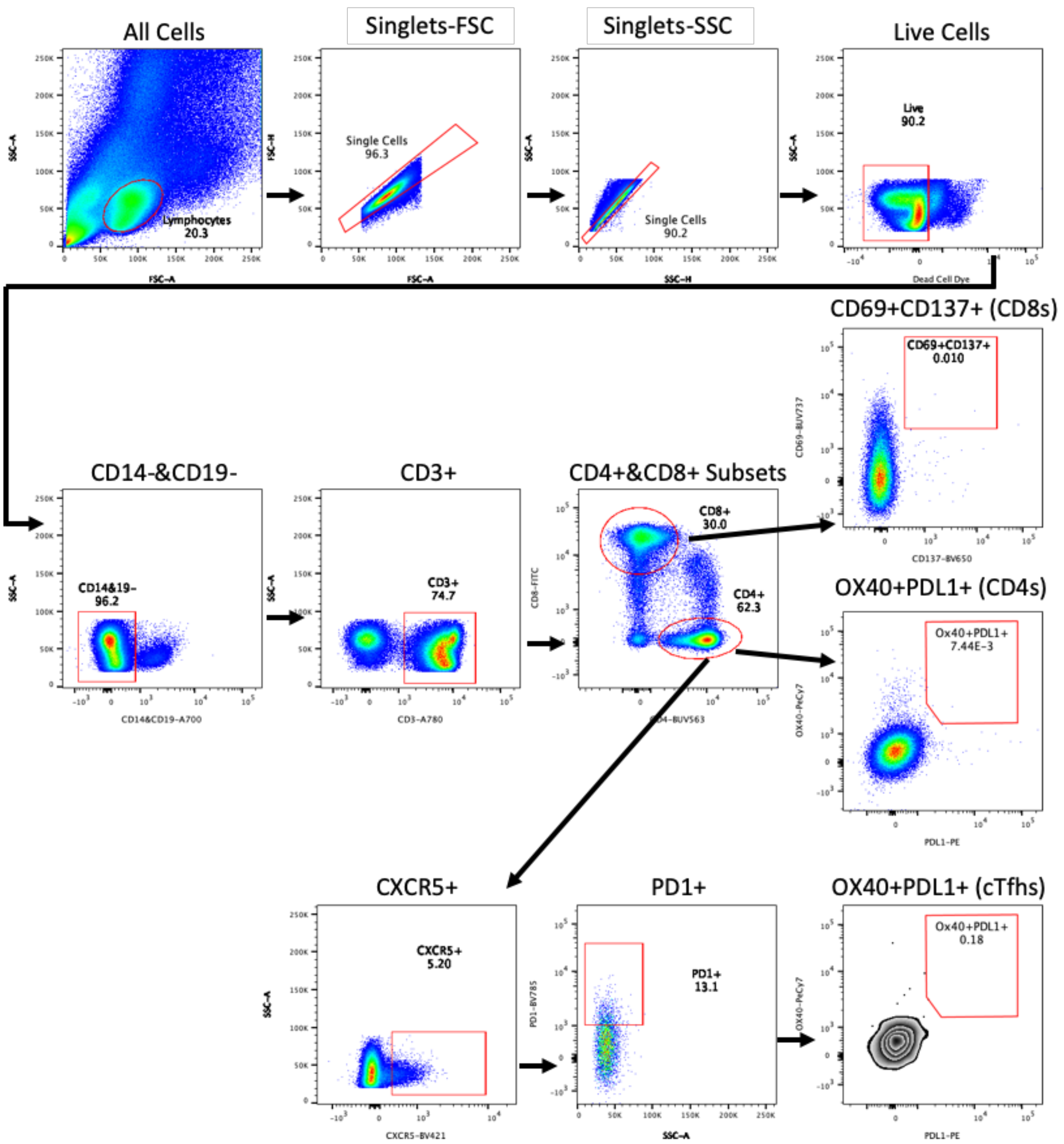

Supplemental Figure 12: Antigen-Specific T-cell Gating Strategy
